# Efficacy of dispirotripiperazine PDSTP in a golden Syrian hamster model of SARS-CoV-2 infection

**DOI:** 10.3389/fmicb.2025.1546946

**Published:** 2025-03-10

**Authors:** Giuseppina Sanna, Olga Riabova, Elena Kazakova, Alexander Lepioshkin, Natalia Monakhova, Alessandra Marongiu, Gianluigi Franci, Aldo Manzin, Vadim Makarov

**Affiliations:** ^1^Department of Biomedical Sciences, Microbiology and Virology Unit, University of Cagliari, Cittadella Universitaria, Cagliari, Italy; ^2^Federal Research Centre “Fundamentals of Biotechnology” of the Russian Academy of Sciences (Research Centre of Biotechnology RAS), Moscow, Russia; ^3^Department of Medicine, Surgery and Dentistry "Scuola Medica Salernitana", University of Salerno, Baronissi, Italy

**Keywords:** SARS-CoV-2, dispirotripiperazine, *in vitro*, hamster model, adhesion, efficacy

## Abstract

The increasing incidence of viral pandemics calls for new small-molecule therapeutics beyond traditional approaches and targets. Dispirotripiperazine, composed of two positively charged nitrogen atoms, represents an unusual scaffold in drug discovery campaigns, and molecules based on it are known to prevent virus infection by disrupting early host–pathogen interactions. In this study, the adhesion-blocking dispirotripiperazine core compound PDSTP was evaluated against SARS-CoV-2 *in vitro* and *in vivo*. We demonstrated that the molecule was acceptably active against two clinical isolates affecting the early stages of the SARS-CoV-2 cycle. In a hamster model of SARS-CoV-2 pneumonia, PDSTP treatment resulted in reduced viral loads in the lungs and turbinates and milder lung tissue lesions. Overall, these data support PDSTP as a preclinical candidate for the treatment of COVID-19.

## 1 Introduction

Over the past two decades, in addition to seasonal flu, there have been numerous outbreaks and pandemics caused by various new viruses. The recently identified severe acute respiratory syndrome-associated coronavirus-2 (SARS-CoV-2) has spread rapidly around the world and led to the coronavirus disease-2019 (COVID-19) pandemic (World Health Organization, 2023; Center for Disease Control and Prevention, 2023). This virus belongs to the *Betacoronavirus* genus, which includes other clinically important coronaviruses, such as SARS-CoV and MERS-CoV (Chen et al., 2020; Perlman and Netland, 2009; Al-Tawfiq and Memish, 2014). The accelerating high mortality rates have bared numerous global public health concerns, including the complete failure of health services to respond to such challenges and the severe shortage of effective antiviral agents. During the SARS-CoV and MERS-CoV outbreaks, an old broad-spectrum antiviral ribavirin and other repurposed compounds were used as therapeutic options, but they did not significantly affect the clinical outcomes (Zumla et al., 2016). Thus, at the onset of the pandemic, there were no specific anti-SARS-CoV-2 drugs or antiviral reserve drugs. Currently, only four small molecules—remdesivir (Veklury), baricitinib (Olumiant), nirmatrelvir in combination with ritonavir to enhance its pharmacokinetics (Paxlovid), and molnupiravir (Lagevrio)—are approved for use for COVID-19 by the U.S. Food & Drug Administration (2023). However, original compounds demonstrating novel mechanisms of action are needed to expand the narrow pipeline of small molecules capable of inhibiting circulating and emerging life-threatening viruses.

In investigating the mechanism of SARS-CoV-2 infection, Hoffmann et al. (2020) along with Letko et al. (2020) revealed that SARS-CoV-2, as well as SARS-CoV, uses the host angiotensin-converting enzyme 2 (ACE2) to bind to and enter cells. However, Clausen et al. (2020) have shown that SARS-CoV-2 first uses polysaccharide residues of heparan sulfate proteoglycans (HSPGs) located on the host cell surface as mediators to bind its spike protein to ACE2 for further membrane fusion. The important role of cellular HSPG in the life cycle of coronaviruses has been confirmed by the observations of other researchers, demonstrating that heparan sulfate facilitates both SARS-CoV and CoV-2 viral spike-mediated entry, as well as SARS-CoV-2 transmission (Kim et al., 2020; Zhang et al., 2020; Chu et al., 2021; Hao et al., 2021; Liu et al., 2021; Bermejo-Jambrina et al., 2021).

Moreover, viruses from other genera also use host cell surface HSPGs to enter cells, including but not limited to herpes simplex virus types 1 and 2, cytomegalovirus, dengue, Marburg and West Nile viruses, chikungunya virus, and respiratory syncytial virus (Liu and Thorp, 2002; Cagno et al., 2019; Koehler et al., 2020; De Pasquale et al., 2021; Chhabra et al., 2021; Hayashida et al., 2022). Thus, heparan sulfate, which serves as an adhesion/attachment factor for a wide range of viruses, is potentially considered a common target for their control.

In this study, we evaluated the efficacy of the viral adhesion-blocking dispirotripiperazine-based molecule PDSTP *in vitro* using a panel of coronaviruses and *in vivo* using a golden Syrian hamster model of SARS-CoV-2-induced pneumonia. Our results indicate that using PDSTP may alleviate viral lung infection in animals.

## 2 Results

Dispirotripiperazines have recently been found to inhibit *in vitro* a range of viruses (Egorova et al., 2021; Schmidtke et al., 2003; Selinka et al., 2007; Paeschke et al., 2014; Adfeldt et al., 2021; Dohme et al., 2022; Alimbarova et al., 2022) by blocking early viral cycle events through binding to negatively charged heparan sulfate sugar residues on HSPGs (Schmidtke et al., 2003; Selinka et al., 2007; Adfeldt et al., 2021). We proposed that dispirotripiperazines may utilize the same target to block SARS-CoV-2 replication, as coronaviruses are also known to use HSPGs to interact with host cells (Clausen et al., 2020; Kim et al., 2020; Zhang et al., 2020; Chu et al., 2021; Hao et al., 2021; Liu et al., 2021; Bermejo-Jambrina et al., 2021; Milewska et al., 2014; Lang et al., 2011). To test this hypothesis, we first evaluated the *in vitro* activity of dispirotripiperazines from our in-house chemical library (Supplementary Table S1) against the well-established betacoronavirus HCoV-OC43 in African green monkey kidney (Vero-76) cells. As PDSTP showed the most compelling profile compared to the other compounds in the series, with an IC_50_ value of 0.7 ± 0.4 μM (Supplementary Table S1), it was selected as the primary tool compound for further *in vitro* and *in vivo* investigations. Using the alphacoronavirus strain HCoV-229E and two clinical betacoronavirus isolates—SARS-CoV-2 VR PV10734 and 35245—we next performed the microtetrazolium test (35245) and the plaque reduction assay (HCoV-229E and PV10734) in Vero cells. As shown in Supplementary Table S1, PDSTP demonstrated its remarkable antiviral activity against the OC43 strain and SARS-CoV-2 isolates with EC_50_ values in the low micromolar range (Table 1). No antiviral activity was detected against the alphacoronavirus HCoV-229E when PDSTP was tested using a plaque reduction assay.

**Table 1:** Cytotoxicity and antiviral activity of PDSTP against coronaviruses in Vero E6 cell line.

Cmpd	Cytotoxicity CC_50_, μM	Antiviral activity IC_50_ against coronaviruses, μM			
		HCoV-229E	SARS-CoV-2 clinical isolates		
			VR PV10734	35245	
		PR	PR	MTT	PR
PDSTP	182.7 ± 40.5	> 100.0	1.8 ± 0.3	14.5 ± 3.3	21.4 ± 7.4
HDQ	40.0 ± 5.0	1.6 ± 0.5	2.4 ± 0.7	11.0 ± 2.9	2.3 ± 0.7
RMD	> 100.0	6.0 ± 2.0	1.6 ± 0.3	-	-

HDQ, Hydroxychloroquine; RMD, Remdesivir; PR, Plaque reduction assay; MTT, Microtetrazolium test. Data are means ± standard deviations (SD) from three independent experiments.

**Figure 1:**
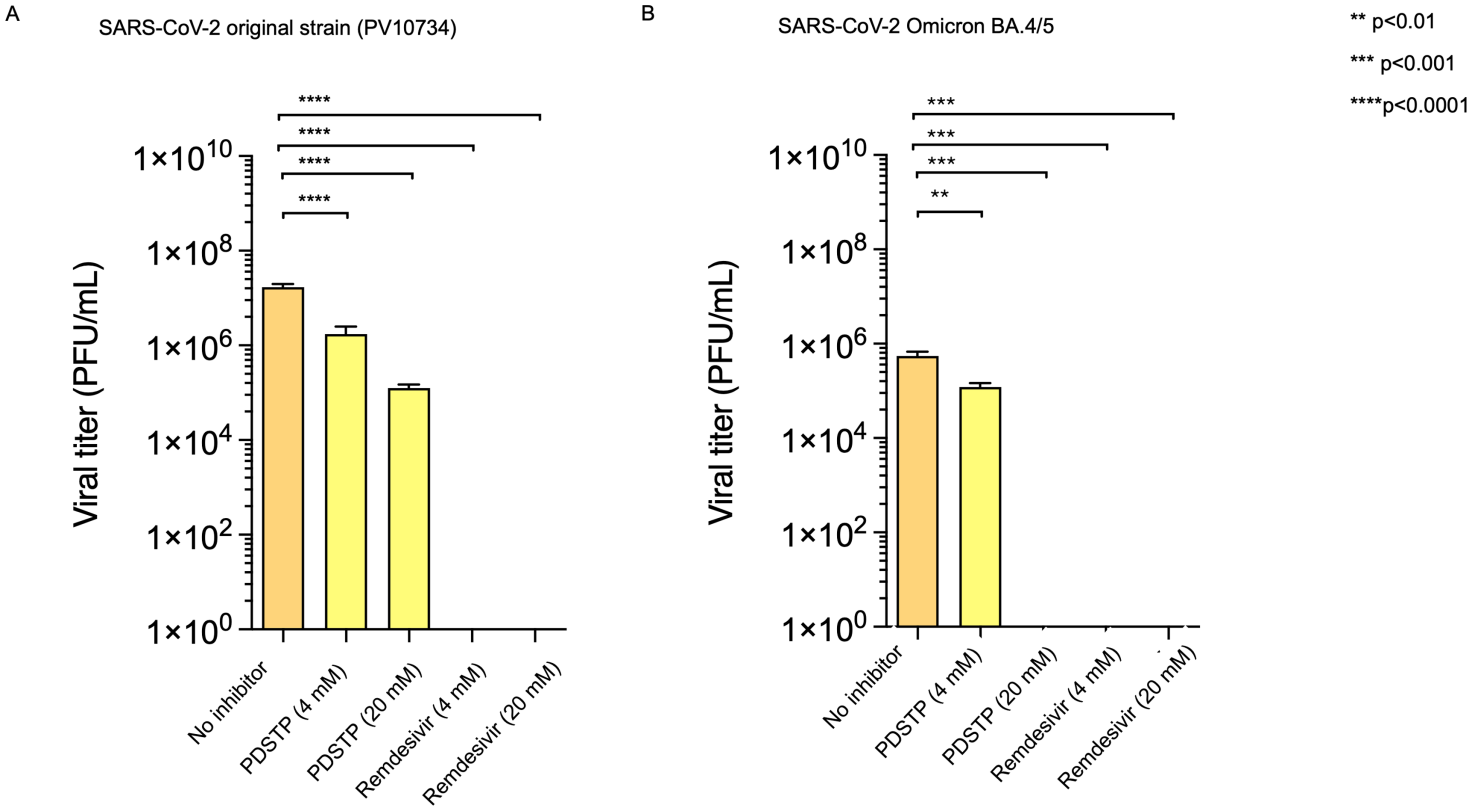
**(A)** Human Calu-3 cells were infected with the SARS-CoV-2 ancestral PV10734 strain and **(B)** BA.4/5 variant (100 PFU). The infected cultures were treated with PDSTP at the indicated doses (20 and 4 μM) or with RDV (20 and 4 μM) as a positive control. Viral yields in the culture supernatant were determined by titration on Vero E6 cells on day 3 post-infection and reported as PFU/mL (mean ± SD). A one-way ANOVA multicomparison was performed. **p < 0.01; ***p < 0.001, ****p < 0.0001.

In our experimental design, we also decided to confirm the data obtained in Vero E6 by testing PDSTP on SARS-CoV-2-Calu-3-infected cells using a yield reduction assay. (Figure 1A). Calu-3 cells were used to determine whether PDSTP affected the yield of infectious virus particles. The results show that PDSTP significantly (****p < 0.0001) reduced the viral titer by approximately two orders of magnitude (100-fold), confirming the inhibitory effect seen in the Vero E6 cell assay.

In addition, PDSTP was also tested on SARS-CoV-2 BA.4/5 variant Calu-3-infected cells, and viral yields were quantified using a plaque assay. Interestingly, the BA.4/5 variant data confirm that PDSTP can strongly reduce viral titers and that its effect is greater than that of the parent strain (Figure 1B).

A virucidal activity assay was performed to evaluate the compound’s effect on SARS-CoV-2 viral infectivity. As expected, no direct impact of PDSTP on viral infectivity was observed when the virus was treated at 4°C and 37°C (Supplementary Figure S1).

**Figure 2:**
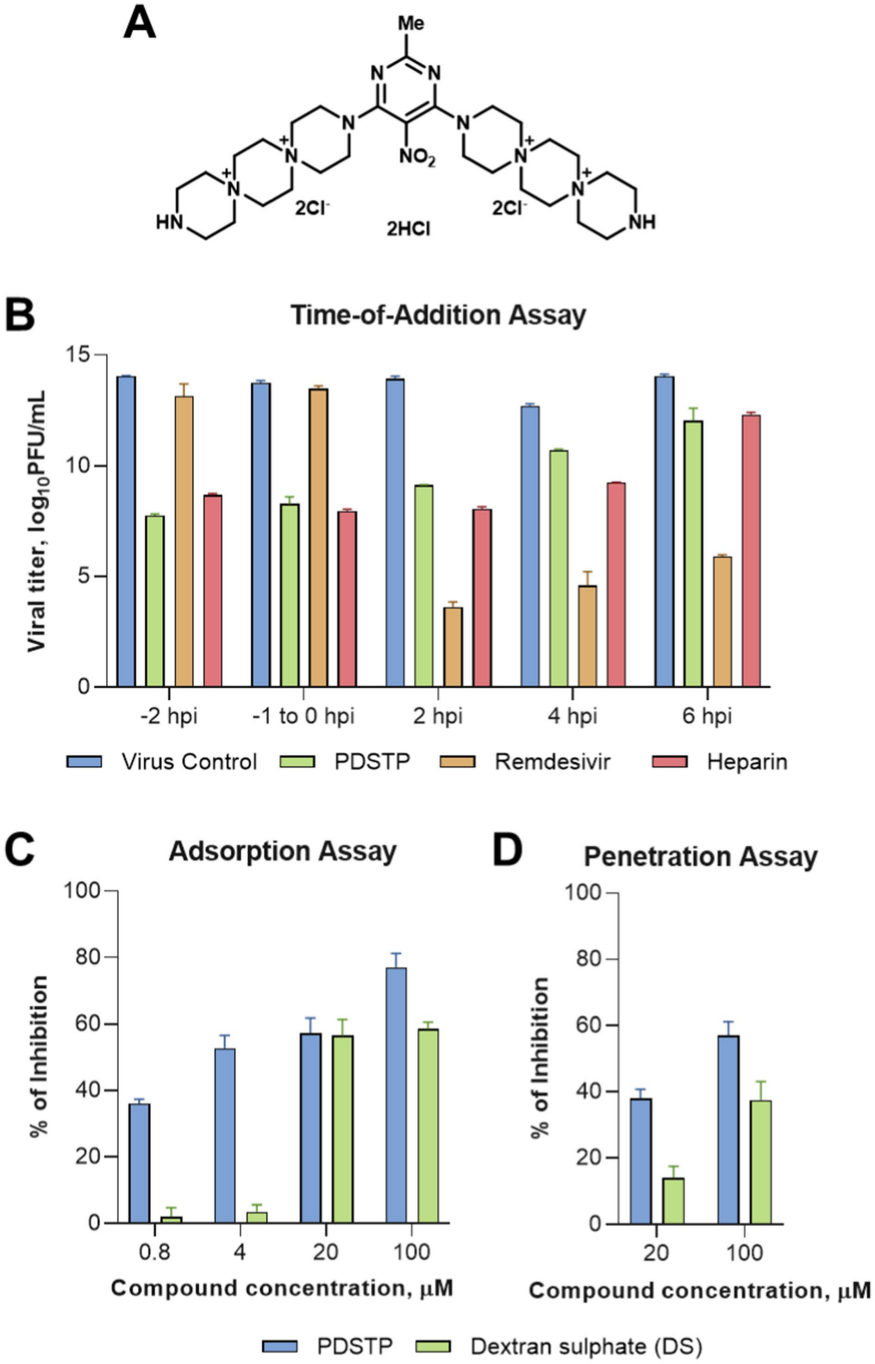
Dispirotripiperazine-based molecule PDSTP and its *in vitro* evaluation. **(A)** Chemical structure of PDSTP; **(B)** in a time-of-addition assay, Vero cells were inoculated with SARS-COV-2 (MOI = 1) and then PDSTP (20 μM) was added at the indicated time points. Viral yields were determined using the plaque assay. Remdesivir and heparin were used as reference compounds; **(C)** the dose-dependent inhibitory effect of PDSTP on SARS-COV-2 adsorption; **(D)** the dose-dependent inhibitory effect of PDSTP on penetration. Dextran sulfate (DS) was used as an internal control. Bars are means and whiskers are standard deviations (SD) from three independent experiments.

To investigate the antiviral mechanism of action of PDSTP, we performed a time-of-drug-addition assay. ToA is an approach routinely used in virology laboratories that can narrow down the target of a newly identified antiviral drug in cell culture by comparing its time of action with that of well-characterized inhibitors. Vero cells were first infected with SARS-CoV-2, and PDSTP, heparin, or remdesivir were added after 0 (during infection), 2, 4, and 6 h post-infection. In addition, a pre-treatment assay was performed to determine whether PDSTP could protect cells from SARS-CoV-2 infection. For this, Vero cells were treated with the compounds for 2 h, and after their removal, the cells were then infected with SARS-CoV-2. Heparin, which acts as a SARS-CoV-2 virion attachment inhibitor (Clausen et al., 2020; Kim et al., 2020; Zhang et al., 2020), and remdesivir, which acts as a nucleoside analog, were used as reference compounds. As shown in Figure 2B, PDSTP exhibited antiviral activity under pre-treatment conditions and in the early stages of infection—during infection (0 h) and immediately after infection (2 hpi)—similar to heparin. As expected, remdesivir did not affect the early events of the viral life cycle. The inhibitory effect was barely noticeable with the addition of PDSTP or heparin at the later stages of infection (4 and 6 hpi), suggesting that PDSTP acts on the early events of the virus life cycle. In contrast, remdesivir exerted its antiviral activity after the SARS-CoV-2 entry process (2, 4, and 6 hpi).

We performed adsorption and penetration assays at a low temperature to further elucidate the early stage of the viral cycle affected by PDSTP (Figures 2C,D). Low-temperature treatment allowed viruses to attach to receptors on the host cell surface but prevented subsequent internalization of the virions. PDSTP treatment led to a dose-dependent reduction of the virus infectivity, showing approximately 57% and 77% viral inhibition at the higher concentrations tested. In contrast, the compound showed only modest viral inhibition at higher concentrations in the penetration experiment, suggesting that PDSTP may interfere with a very early step of the host–pathogen interaction.

To determine the *in vivo* efficacy of PDSTP, a golden Syrian hamster model of SARS-CoV-2-induced pneumonia was used as a suitable animal model for the preclinical evaluation of small molecule anti-coronavirus drugs (Muñoz-Fontela et al., 2020).

**Figure 3:**
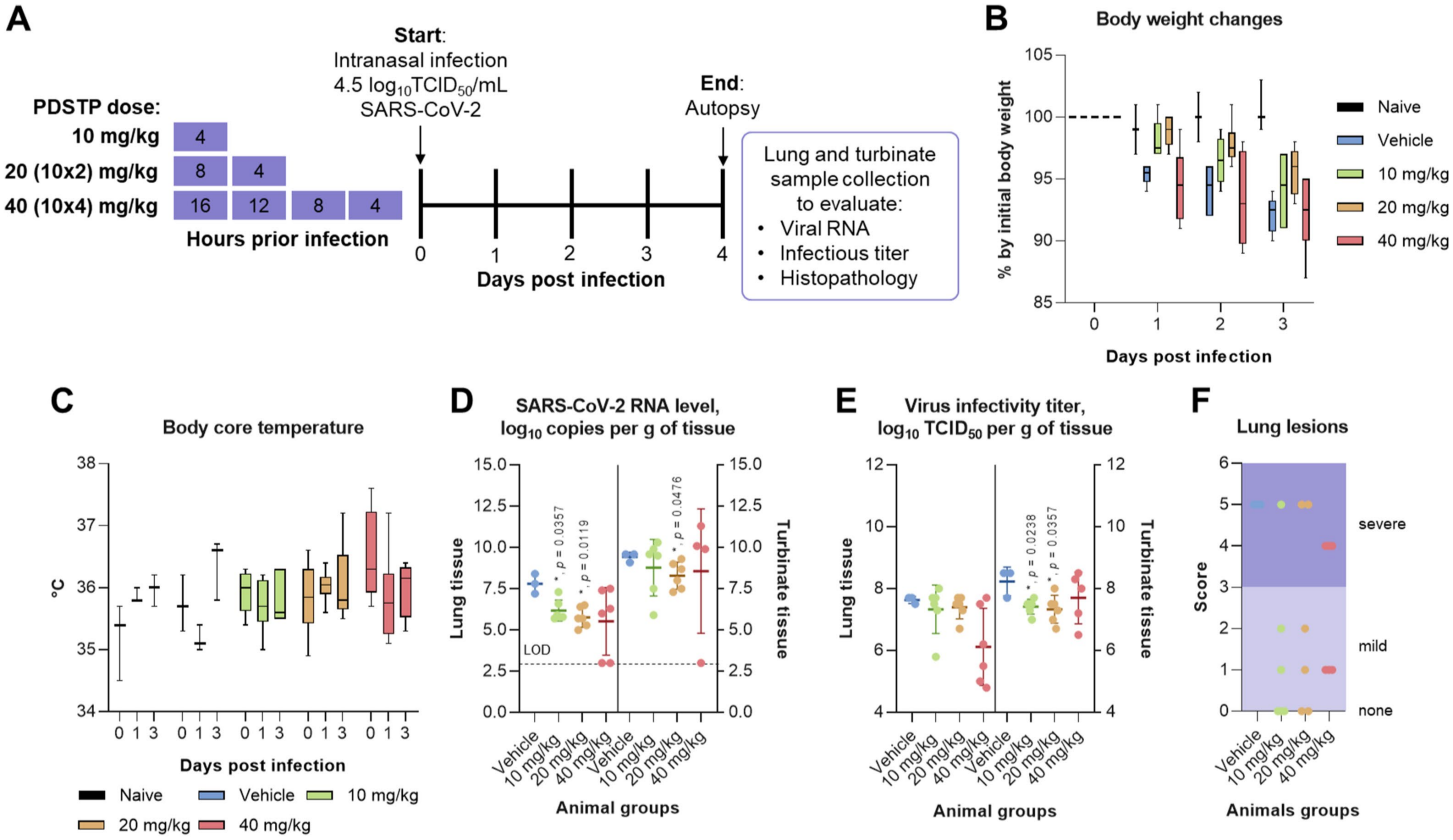
Efficacy of dispirotripiperazine-based molecule PDSTP in a Syrian hamster model of SARS-CoV-2 pneumonia using various drug regimens. **(A)** Efficacy study design. Three animal groups received PDSTP at three doses (10, 20, and 40 mg/kg) intranasally according to the drug scheme, and one animal group was treated with vehicle (0.9% saline solution) (not shown). The animals were monitored for body weight **(B)** and core body temperature **(C)** changes. Immediately after euthanasia, lungs and turbinates were removed to evaluate viral RNA and infectious titer in tissues. SARS-CoV-2 RNA copies **(D)** in lung or turbinate tissue samples were determined using the qRT-PCR and expressed as log_10_ viral RNA copies per gram of tissue. The dashed line indicates the limit of detection (LOD). SARS-CoV-2 infectious titer **(E)** in lung or turbinate tissue samples was measured using the plaque assay and expressed as log_10_ viral TCID_50_ per gram of tissue. **(F)** Lungs from individual animals were examined for the presence (marked as 1, Supplementary Table S1) or absence (marked as 0, Supplementary Table S1) of the following lung lesions: hemorrhage areas, vasculitis, inflammation foci, pulmonary alveolar edema, epithelial injury, and necrosis. The total score is shown for individual animals. For panels **(B,C)**, the boxes encompass the 25^th^ to 75^th^ quartile, the line is the median, and the whiskers are the range. For panels **(D–F)**, dots represent values for individual animals from all groups, the bold line is the mean, and the whiskers are the standard deviation (SD). Statistically significant differences from the vehicle, marked with asterisks, were determined using the Mann–Whitney *U*-test **(D**–**G)**. For all statistical analyses, a *p*-value of ≤0.05 was considered significant.

We administered three doses of PDSTP—10, 20, and 40 mg/kg—intranasally at different intervals before the virus challenge: once, 4 h before the virus challenge (10 mg/kg); twice, 8 and 4 h before infection in equal volumes (20 mg/kg); and four times every 4 h, ending 4 h before the challenge (40 mg/kg), as shown in Figure 3A. Then, the animals were intranasally infected with 4.5 log_10_TCID_50_/mL of SARS-CoV-2 B.1.1. alpha variant. The animals were monitored daily for changes in body weight (Figure 3B) and core body temperature (Figure 3C). While the naïve animals steadily gained body weight, both the PDSTP- and vehicle-treated animals consistently lost body weight, maintaining a loss of no more than 10% until the end of the study. Сore body temperature in all animal groups was within the normal range. None of the animals died.

As shown in Figure 3D, intranasal administration of PDSTP at doses of 10 and 20 mg/kg significantly reduced viral RNA copy levels in lung tissue compared to the vehicle-received group by 1.6 log_10_ and 2.0 log_10_, respectively (*p* = 0.035 and *p* = 0.0119). At the same time, although PDSTP at 40 mg/kg showed a decrease of 2.2 log_10_, and the SARS-CoV-2 RNA yield in two animals from the group was not determined, this decrease was statistically non-significant. In the upper respiratory tract (turbinate tissue), viral RNA copy levels were statistically significantly diminished only in animals treated with 20 mg/kg of PDSTP (*p* = 0.0476). At 40 mg/kg, PDSTP demonstrated a 1.5 log_10_ reduction in lung viral titer compared to the negative control, but this was not statistically significant due to the small group size (Figure 3E). In the nasal tissue, PDSTP at doses of 10 and 20 mg/kg reduced viral titers (~ 1.0 log_10_) compared to the vehicle group (*p* = 0.0238 and *p* = 0.0357); however, the reduction at 40 mg/kg was not statistically significant.

**Figure 4:**
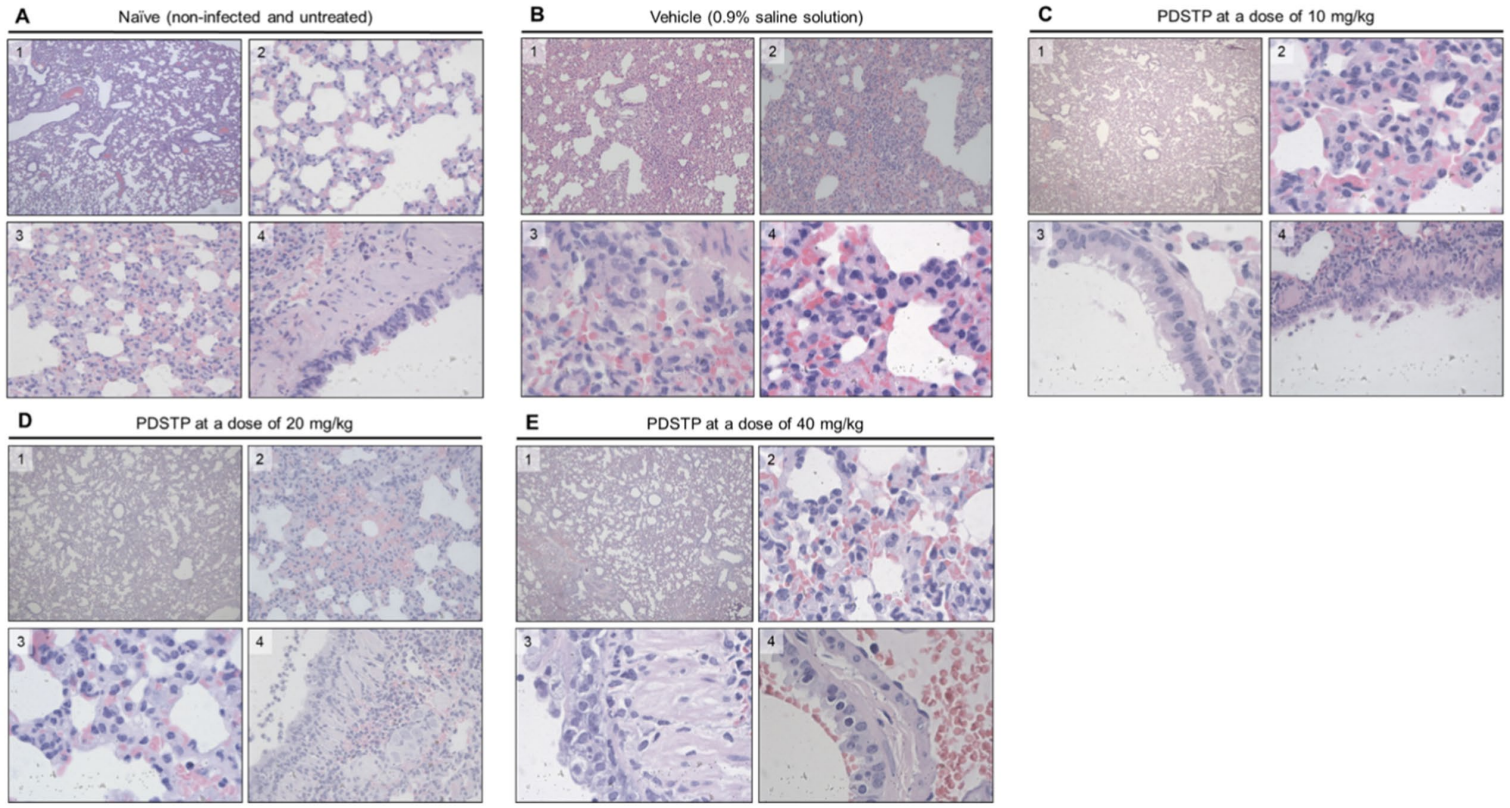
Hematoxylin and eosin (H&E) staining was used to examine the pathology of lung sections from treated animals. Representative images are shown. **(A)** Naïve animals; 1, objective ×10; 2, objective ×40; 3, objective ×100, 4, objective ×40; **(B)** animals received 0.9% saline solution: 1, objective ×4; 2, objective ×20; 3 and 4, objective ×100; **(C)** animals treated with 10 mg/kg of PDSTP: 1, objective ×4; 2 and 3, objective ×100; 4, objective ×40; **(D)** animals treated with 20 mg/kg of PDSTP: 1, objective ×4; 2, objective ×40; 3, objective ×100; 4, objective ×40; **(E)** animals treated with 40 mg/kg of PDSTP: 1, objective ×4; 2, objective ×1,000; 3 and 4, objective ×400.

Lung samples were collected for histopathological analysis, and the results are shown in Figure 4 (and detail in Supplementary material). The overall SARS-CoV-2 infection severity score was calculated from 0 to 6. The results for individual animals are shown in Figure 3F. Treatment improved virus-induced lung pathology, particularly at the 10 mg/kg dose, with lung severity scores of “none” in three out of six animals and “mild” in two animals (Figure 3F). Histopathological examination in untreated animals revealed some features of pneumonia induced by the SARS-CoV-2 virus. These included acute alveolar edema with inflammatory infiltrates containing accumulations of foamy intra-alveolar macrophages, microxyphil leukocytes, and mononuclear cells (Figure 4A). Bronchial damage, including multifocal exfoliation of epithelial cell sloughing, necrosis, and hemorrhage, was also observed. In animals treated with either 10 or 20 mg/kg PDSTP, alveolar edema was moderate, with a reduced number of inflammatory infiltrate cells (Figures 4B,C).

## 3 Materials and methods

### 3.1 Biosafety and ethics statement

All work with infectious SARS-CoV-2 was performed in biosafety level-3 facilities at the Department of Biomedical Sciences of the Cagliari University (Sardinia, Italy) for *in vitro* assays and at the Mechnikov Research Institute for Vaccines and Sera (Moscow, Russia) for *in vivo* experiments.

An efficacy study was carried out under protocols approved by the Animal Care and Use Committee at the Mechnikov Research Institute for Vaccines and Sera (Moscow, Russia) according to the institute’s guidelines for animal use, the state industry standards GOST 33215-2014 and 33216-2014, the European Directive 2010/63/ЕС, and the Guide for the Care and Use of Laboratory Animals, 8th edition (Washington, DC: National Academies Press; 2011).

### 3.2 Cell lines

African Green Monkey (*Cercopithecus aethiops*) kidney Vero-76 (CRL-1587), Vero E6 (CRL-1586), human lung adenocarcinoma Calu-3 (ATCC HTB-55), and human lung fibroblasts MRC-5 (CCL-171) cell lines were purchased from the American Type Culture Collection (ATCC), and the Vero (cat# 1.5.1.1) cell line was purchased from the Ivanovsky Institute of Virology Collection (as a division of the Gamaleya National Research Centre for Epidemiology and Microbiology, Moscow, Russia). Cell cultures were routinely checked for mycoplasma contamination using a Hoechst staining kit (MP Biomedicals, LLC, Santa Ana, CA, United States).

### 3.3 Virus strains

The HCoV-OC43 (VR-1558) strains and the HCoV-229E (VR-740) strains were purchased from the ATCC and propagated on Vero-76 and MRC-5 cells, respectively. The SARS-CoV-2 PV10734 Italian strain (D614G, lineage B.1.1), provided by the Lazzaro Spallanzani Hospital (Rome, Italy), and the SARS-CoV-235245 clinical isolate (PIK35, lineage B.1.1), provided by the Research Institute of Influenza (St. Petersburg, Russia), were propagated on Vero E6 cell culture. The SARS-CoV-2 BA4/5, VRPA58032 variant, was provided by Professor G. Giammanco, University of Palermo, Italy. The SARS-CoV-2 B.1.1 GR MM1/2020/Aug-21 clinical isolate was propagated on Vero cell culture.

### 3.4 Compounds and chemicals

PDSTP (3,3′-(2-methyl-5-nitropyrimidine-4,6-diyl)bis-3,12-diaza-6,9-diazonia-dispiro[5.2.5.2]hexadecane tetrachloride dihydrochloride nonahydrate) was synthesized with minimal changes, as described previously (Schmidtke et al., 2002), and its purity was determined using the HPLC analysis (Supplementary Figure S2). Remdesivir (RDV) and hydroxychloroquine (HDQ) were purchased from MedChemExpress (Monmouth Junction, NJ, United States) and used as positive controls for *in vitro* studies. Dextran sulfate (DS) was purchased from Sigma-Aldrich (MO USA).

### 3.5 *In vitro* antiviral activity assays

#### 3.5.1 MTT test

The compound’s activity against OC43 and SARS-CoV-235245 was based on the inhibition of virus-induced cytopathogenicity in acutely infected Vero-76 cells (MOI 0.01). Briefly, Vero-76 cells were seeded in 96-well plates at a density of 3 × 10^4^ cells/well and incubated in a growth medium in a 5% CO_2_ at 37°C overnight. Cell monolayers were then infected with 50 μL of the appropriate viral dilution in maintenance medium [D-MEM and MEM-Earl (Gibco, Thermo Fisher Scientific, MA United States) with L-glutamine (Gibco), 1 mM sodium pyruvate (Gibco), and 0.025 g/L kanamycin (Gibco), supplemented with 0.5% inactivated FBS (Harlan Sera-Lab, Loughborough, United Kingdom), respectively] to give an MOI of 0.01, without or with serial dilutions of test samples. After 6 (HCoV-OC43) and 4/5 (SARS-CoV-2) days of incubation at 37°C, the extent of cell viability was quantified using a tetrazolium-based colorimetric method, in which the reduction of 3-(4,5-dimethylthiazol-2-yl)-2,5-diphenyl-tetrazolium bromide (MTT) by mitochondrial dehydrogenases to a soluble colored formazan was measured using a spectrophotometer (Infinity 200, Tecan Austria GmbH, Austria) at 570 nm, as previously reported (Sanna et al., 2020).

#### 3.5.2 Plaque reduction assay

Vero E6 cells were incubated in the presence of PDSTP and reference compounds (100.0, 20.0, 4.0, and 0.8 μM concentrations) for 1 h at 37°C in a 5% CO_2_ and then infected for 1 h with 250 μL of the corresponding virus (SARS-CoV-2 PV10734 or HCoV-229E) dilutions to obtain 50–100 PFU/well. The plates were then incubated for 1 h at 37°C in a 5% CO_2_ atmosphere. Following the removal of the unabsorbed virus, 400 μL of the medium (D-MEM with L-glutamine, 4,500 mg/L D-glucose, and 1% inactivated FBS) containing 0.75% methyl-cellulose (M0512, viscosity 4,000 cP, Sigma-Aldrich, United States) and the serial dilutions of the compounds were added and incubated for 4 (SARS-CoV-2) or 2 (HCoV-229E) days at 37°C in a 5% CO_2_ atmosphere. Subsequently, the cells were fixed and stained with a 0.2% crystal violet solution (V5265, Sigma-Aldrich, United States) and then the number of plaques was calculated. The degree of cell growth/viability and viral multiplication at each drug concentration tested was expressed as a percentage of the untreated control. Concentrations resulting in 50% inhibition (CC_50_ or EC_50_) were determined using linear regression analysis.

#### 3.5.3 Yield reduction assays

Calu-3 cells (5 × 10^5^/mL) were infected with SARS-CoV-2, ancestral strain, and BA.4/5 variant (100 PFU/mL) in the maintenance medium and contemporary PDSTP, respectively. RDV, as a reference compound, was tested. After 72 h at 37°C and 5% CO_2_, each sample was harvested and stored at −80°C. The samples were then diluted with serial passages, starting from 10^−1^ to 10^−10^. The titer of the virus-containing supernatant dilutions series was determined using the plaque assay in Vero cells.

### 3.6 *In vitro* mechanism of action study

#### 3.6.1 Cell pre-treatment assay

Vero cells in 24-well plates were incubated with a 20 μM concentration of PDSTP or remdesivir for 2 h. After the compounds were removed, the cells were infected with SARS-CoV-2 PV10734. After virus adsorption to the cells, the inoculum was removed and the cells were then overlaid with medium and incubated for 96 h at 37°C. The virus titers were then determined using the plaque assay.

#### 3.6.2 Time-of-addition assay (ToA)

The confluent monolayers of Vero cells in 24-well tissue culture plates were infected for 1 h at room temperature with SARS-CoV-2 PV10734 dilutions to give a final MOI of 1.0. After adsorption, the monolayers were washed two times with DMEM medium and incubated with the same medium at 5% CO_2_ and 37°C (time zero). Monolayers were treated with PDSTP (20 μM, approximately 10 times higher than the IC_50_) or reference for 1 h during the infection period (at −1 to 0) and at specific time points, 2, 4, and 6 h post-infection (This approach determines how long the addition of a compound can be postponed before it loses its antiviral activity.) After each incubation period, the monolayers were washed two times with maintenance medium and incubated with the fresh medium until 15 h post-infection (Daelemans et al., 2011).

Monolayers obtained from the ToA assay were frozen at −80°C to arrest ongoing infection. After the freeze–thawing procedure, the samples were collected and centrifuged, and the viral titers were determined using the plaque assay. Data were then log-transformed, and the results were presented as means ± standard deviation (SD).

#### 3.6.3 Adsorption assay

Vero cells grown in 24-well plates were infected with SARS-CoV-2 PV10734 (MOI 0.1) in the presence or absence of PDSTP and incubated for 2 h at 4°C. The medium containing the un-adsorbed virus was then removed. The cells were washed twice with PBS and overlaid with a fresh medium. Plaques were counted after 96 h of incubation at 37°C. Dextran sulfate (DS) was used as an internal control.

#### 3.6.4 Penetration assay

A penetration assay was performed with small modifications according to the method reported earlier (Chang et al., 2012). A 24-well tissue culture plate was seeded with Vero cells (3 × 10^5^ cells/well), which were then incubated overnight at 37°C in a 5% CO_2_ atmosphere. The cells were pre-chilled on ice for 1 h, and the medium was removed. The cells were infected with 100 PFU of SARS-CoV-2 PV10734 on ice for 2 h. The medium containing the unbound virus was then removed. Various concentrations of PDSTP (100–0.8 μM) in the medium were added, and the cells were incubated at 37°C for 1 h to trigger endocytosis of the virus. The infected cells were then treated with alkaline PBS (pH 11) for 1 min to inactivate viruses that had not penetrated the cells. Acidic PBS (pH 3) was then added to neutralize the mixture. The neutralized medium was removed; the cells were overlaid with methylcellulose in media and then were incubated at 37°C. After the 72-h incubation period, the cells were stained, and the plaques were determined by counting. Dextran sulfate (DS) was used as an internal control.

### 3.7 Efficacy study

#### 3.7.1 Animals

Thirty 9- to 10-week-old male golden Syrian hamsters weighing 90.0 ± 10.0 g were purchased from the Scientific Production Association “Home of Pharmacy” (Russia). All animals were kept in cages under standard laboratory conditions (temperature 20 ± 2°C, 12-h light/dark cycle) and were fed standard rodent compound feed “LBK-120” (Tosnensky Mixed Feed Factory, Russia) twice a day, with water provided *ad libitum*. The animals were off-feed for 1 h with free access to water before the intranasal challenge.

#### 3.7.2 Experiment design

Male superficially anesthetized [Zoletil 100 (Virbac, France), 40 mg/kg, intramuscular administration] hamsters were randomly divided into five groups (*n* = 6). The first animal group was healthy animals (naïve control). The second group received an intranasal vehicle (0.9% saline solution) used as a negative control. PDSTP was administered intranasally as a 2.5% aqueous solution using a manual pipette (Thermo Fisher Scientific, MA, United States) according to the schedule shown in Figure 3A. The animals were then intranasally infected with 4.5 log_10_TCID_50_/mL (13 μL per nostril) of SARS-CoV-2 B.1.1 GR MM1/2020/Aug-21 clinical isolate. Body weights were monitored daily, and changes were calculated as the ratio of daily body weight to initial body weight and expressed as a percentage. Core body temperature was measured using a MicroTherma 2 T thermometer equipped with a RET-3 small animal rectal probe (ThermoWorks, UT, United States). Core body temperature measured before intranasal infection on day 0 was used as baseline temperature. All animals were euthanized on 4 dpi with an overdose of a combination of injectable anesthetics Zoletil 100 (50 mg/mL) and Xyla (20 mg/kg) (Interchemie Werken De Adelaar B.V., Netherlands) at a dose of 1.0 mL per kg of body weight, and the lungs were removed for further analysis.

#### 3.7.3 Lung pathology examination

Macroscopic visual examination was performed using a Nikon Coolpix W300 digital camera (Nikon Corporation, Japan). Lung lesions were then calculated as a percentage of the total lung surface. For histological examination, the left lung was fixed in 30 mL of 10% neutral buffered formalin HistoPot (Serosep, Ireland) and embedded with paraffin. The tissue was cut into sections with a thickness of 3–4 μm, and the slices were then stained with hematoxylin and eosin. Histological examination was performed using a Leica DM6000 microscope (Leica Microsystems, Germany), and photomicrographs were taken using Nikon DS (Nikon) and Leica DFC 490 (Leica) digital cameras for microscopy. The right lung was then used for further analysis.

#### 3.7.4 SARS-CoV-2 RT–qPCR assay

Immediately after weighing the right lung, the caudal lobe of the right lung was submerged in RNAprotect Tissue Reagent (Qiagen, Germany) at 4°C for 24 h. The RNAprotect Tissue Reagent was removed, and the QIAzol Lysis Reagent (900 μL) from the RNeasy Plus Universal Mini Kit (Qiagen) was added to the lung tissue. The tissue was homogenized using a FastPrep-24 5G homogenizer system (MP Biomedicals, CA, United States) at 6.0 m/s for 40 s and frozen at −80°C. RNA was isolated using the RNeasy Plus Universal Mini Kit, according to the manufacturer’s protocol. The RNA concentration and quality in each sample (2.0 μL) were evaluated using a NanoDrop 1000 spectrophotometer (Thermo Fisher Scientific). The ratio of absorbance at 260 nm and 280 nm is used to assess the purity of RNA. A ratio of 1.9–2.0 is generally considered “pure” for RNA, according to the manufacturer’s manual.

A measure of 2 μL of a viral RNA sample was used for RT–qPCR to detect and quantify the SARS-CoV-2 N gene using TaqMan Fast Virus 1-Step Master Mix (Thermo Fisher Scientific). Each RNA sample was tested in triplicate using a CFX96 Touch Deep Well Real-Time PCR detection system (Bio-Rad Laboratories, Hercules, CA, United States). Viral copies were calculated using a viral RNA standard curve obtained with serial 10-fold dilutions (10^1^–10^6^) of RNA, and primers and probes targeting the SARS-CoV-2 N gene: 5′-CACATTGGCACCCGCAATC-3′ (forward), 5′-GTTCCTTGTCTGATTAGTTCCTGGT-3′ (reverse), and 5′-FAM-TTCTACGCAGAAGGGAGCAGAGGC-BHQ1-3′ (probe). The primer and probe sequences for the hamster *β*-2-microglobulin gene used as a reference are 5′-GGCTCACAGGGAGTTTGTAC-3′ (forward), 5′-TGGGCTCCTTCAGAGTTATG-3′ (reverse), and 5′-VIC-CTGCGACTGATAAATACGCCTGCA-BHQ1-3′ (probe). To generate a standard curve for the SARS-CoV-2 N gene RNA assay, the SARS-CoV-2 N gene RNA was synthesized using the pUC57:N-gene plasmid (Innova Plus, Russia). Transcribed RNA was purified using the RNeasy Plus Universal Mini Kit according to the manufacturer’s protocol, and serial 10-fold dilutions (10^1^–10^6^) were prepared and used to generate the standard curve. The limit of detection (LOD) was 10^3^ copies/g.

#### 3.7.5 Lung SARS-CoV-2 virus titer determination

To determine SARS-CoV-2 infectious titer, the cranial and middle lobes of the right lung were used. To prepare lung homogenates, the tissue samples were homogenized in Eagle’s minimal essential medium (EMEM) supplemented with 2% fetal bovine serum and 1% penicillin/streptomycin in a proportion 1:10 m/v (Gibco, Thermo Fisher Scientific) at 4.0 m/s for 30 s. The homogenate was then centrifuged at 3500 rpm and 4°C for 10 min, and 100 μL of the supernatant in 900 μL of EMEM was filtered through a syringe filter with 0.22-μm pore size. 96-well plates containing Vero cells were inoculated with 100 μL per well of serial dilutions of each sample (10^−2^–10^−8^). Each dilution was performed in quadruplicate. After 3 days of incubation at 37°C in a 5% CO_2_ atmosphere, the plates were analyzed for the absence or presence of cytopathic effect in each well using an AE31 Elite 30 W trinocular inverted microscope (Motic, China). Infectious titers as log_10_ TCID_50_ per g of lung tissue were calculated using the Reed and Muench (1938) method. The limit of detection (LOD) was 100 copies/g.

#### 3.7.6 Statistical analysis

All experiments were performed in triplicate. All data are expressed as the mean ± standard deviation (less than 15% if not reported). Excel and GraphPad Prism software were used to perform all statistical data analyses. Statistical significance was determined using the non-parametric Mann–Whitney *U-*test. A one-way ANOVA was used to analyze *in vitro* antiviral data. For all statistical analyses, a *p*-value of ≤ 0.05 was considered significant.

## 4 Discussion

In this study, we report the promising *in vitro* anti-*β*-coronavirus activity and *in vivo* efficacy in a model of SARS-CoV-2 pneumonia of the dispirotripiperazine core derivative PDSTP, an original low-toxic small molecule with a good safety preclinical profile (Supplementary material).

Among the small-molecule COVID-19 antivirals, molnupiravir, a nucleoside analog of viral RNA-dependent polymerase, and nirmatrelvir, a SARS-CoV-2 protease inhibitor, in combination with ritonavir have been approved for emergency use to treat high-risk COVID-19 patients (Sidebottom et al., 2021). These drugs selectively utilize these targets to inhibit viral replication, and SARS-CoV-2 with a short replication time can frequently produce escape mutations that confer drug resistance, so a combination of several antiviral drugs may be required for its effective treatment (Markov et al., 2023). Therefore, an alternative to available antivirals may be an inhibitor that targets an early stage of the viral life cycle. Among the various approaches, prevention of viral adsorption by blocking cell surface heparan sulfate proteoglycans offers a good opportunity to treat SARS-CoV-2 infection in combination with traditional antiviral agents (Clausen et al., 2020; Zhang et al., 2020).

Dispirotripiperazines are dispiro compounds containing three-cycle chemical moieties that share two positively charged nitrogen atoms (Figure 2A; Supplementary Table S1) (Egorova et al., 2021; Schmidtke et al., 2003; Selinka et al., 2007; Paeschke et al., 2014; Adfeldt et al., 2021; Dohme et al., 2022; Alimbarova et al., 2022) that are known to use HSPGs to adsorb and enter host cells (Kim et al., 2020; Zhang et al., 2020; Chu et al., 2021; Hao et al., 2021; Liu et al., 2021; Bermejo-Jambrina et al., 2021).

Moreover, PDSTP has shown efficacy in a rabbit model of herpes simplex epithelial keratitis, reducing the mean progression and corneal lesions comparable to traditional aciclovir (Alimbarova et al., 2022). The present study complements and extends other work using cell surface heparan sulfate residues targeting as a non-standard therapeutic strategy to inhibit SARS-CoV-2 and other life-threatening viruses (Clausen et al., 2020; Zhang et al., 2020). PDSTP showed no *in vitro* activity against the alphacoronavirus HCoV-229E but showed potent activity against the betacoronaviruses tested, HCoV-OC43 and SARS-CoV-2 (Table 1 and Supplementary Table S1). These alpha- and betacoronaviruses bind different host cell receptors (Chen et al., 2020; Perlman and Netland, 2009). While in OC43 and SARS-CoV-2, the interaction of the S protein with cell surface HSPGs triggers a conformational change in the receptor binding domain (RBD) of the S protein, facilitating the binding of the virus to its specific receptor (ACE) (Hoffmann et al., 2020; Letko et al., 2020; Clausen et al., 2020; Kim et al., 2020; Zhang et al., 2020; Chu et al., 2021; Hao et al., 2021; Liu et al., 2021; Bermejo-Jambrina et al., 2021), there is no mechanistic evidence suggesting that these cell surface sugar residues are required for 229E attachment and infection of target cells (Hu et al., 2021; Hu et al., 2022). Vero E6 and Calu-3 cells have been widely used as model substrates for SARS-CoV-2 *in vitro* assays due to their susceptibility to the virus. However, several studies have described a selective pressure on SARS-CoV-2 during propagation in Vero E6 cells, leading to mutations mainly at the furin cleavage site of the viral genome as it adapts to the absence of human TMPRSS2 in these cells (Matsuyama et al., 2020; Klimstra et al., 2020). Indeed, the data that have been published so far are not consistent, and some publications do not detect these described mutations at the furin cleavage site when SARS-CoV-2 is grown on Vero E6 for four or even ten passages. However, the exact reason for these irregularly observed mutations is still under debate. In some publications, these described mutations at the furin cleavage site are not detected when SARS-CoV-2 is grown on Vero E6 for several passages (Harcourt et al., 2020) However, the exact cause of these irregularly observed mutations is still under debate (Mautner et al., 2022). To overcome this problem, virus isolation and propagation could be performed on Vero E6, while infection studies or drug screening could be performed on the human cell lines Caco-2 and Calu-3.

For this reason, PDSTP was also evaluated using a yield reduction assay on Calu-3 infected with the parent strain and the BA.4/5 variant. The results show that PDSTP significantly reduces viral titers, confirming the inhibitory effect seen in the Vero E6 cell assay. The results of the virucidal activity assay (Supplementary Figure S1) showed that PDSTP had no direct effect on SARS-CoV-2. ToA is an approach routinely used in virology laboratories that can narrow down the target of a newly identified antiviral drug in cell culture by comparing its time of action with that of well-characterized inhibitors.

This mechanism of action study supported the antiviral effect of PDSTP only at the early stages of viral infection (Figures 2B,C) in a heparin-like manner. This result is consistent with the previous experimental results that heparin inhibits SARS-CoV-2 through competitive binding to host surface heparan sulfate proteoglycans (Clausen et al., 2020; Zhang et al., 2020; Mycroft-West et al., 2020; Tandon et al., 2021; Paiardi et al., 2022).

Taken together, our results suggest that PDSTP may target cell surface heparan sulfate residues to display its antiviral activity.

In an *in vivo* study, we observed a positive effect of PDSTP on SARS-CoV-2-infected animals based on collective results. Lower levels of SARS-CoV-2 RNA in the lungs and turbinates were detected in animals treated with PDSTP compared to infected animals. At the same time, a statistically significant decrease in SARS-CoV-2 infectious titer in PDSTP animal groups was observed only in the turbinates, not in the lungs. We hypothesized that this finding may be associated with the difficulty of stable injection of PDSTP directly into the lungs, requiring further investigation and application of aerosol devices. However, the beneficial effect of PDSTP was also supported by histopathological examination [A detailed description and comprehensive analysis of the histological images is provided in Supplementary material (“Histological Description”)] PDSTP at doses of 10 or 20 mg/kg completely reduced SARS-CoV-2-induced lung pathology in three and two animals, respectively, and alleviated the condition in two animals, whereas only severe pneumonia was observed in untreated animals. Although we observed high variability in treatment outcomes, when hamsters were treated with PDSTP at a dose of 40 mg/kg, two animals from this group showed a strong decrease in viral RNA yield below LOD and an infectious titer in the lungs. As mentioned above, we were disappointed with the data on the reduction of lung viral titers, but the beneficial effect of PDSTP is evident and confirmed by other data. This suggests that, in addition to the antiviral effect described in this article, other factors also contribute to the multiple effects of PDSTP, playing an important role in its overall efficacy. We cannot exclude the antimicrobial effect of PDSTP described in a previous study (Bonacorsi et al., 2024), which also seems to be realized in this case and leads to an overall positive effect of PDSTP on the course of the disease.

Overall, in this study, we provide proof-of-concept for using an adhesion-blocking molecule as a promising strategy to treat SARS-CoV-2 infection. Together with our previous findings showing that this compound inhibits various viruses that use heparan-sulfate proteoglycans to interact with host cells, we report that this approach will go beyond the traditional “one disease-one target-one drug” strategy used in current antiviral strategies.

## Data Availability

The raw data supporting the conclusions of this article will be made available by the authors, without undue reservation.

## References

[ref1] Adfeldt R. Schmitz J. Kropff B. Thomas M. Monakhova N. Hölper J. E.. (2021). Diazadispiroalkane derivatives are new viral entry inhibitors. Antimicrob. Agents Chemother. 65:e02103. doi: 10.1128/AAC.02103-20, PMID: 33495228 PMC8097465

[ref2] Alimbarova L. Egorova A. Riabova O. Monakhova N. Makarov V. (2022). A proof-of- concept study for the efficacy of dispirotripiperazine PDSTP in a rabbit model of herpes simplex epithelial keratitis. Antivir. Res. 202:105327. doi: 10.1016/j.antiviral.2022.105327, PMID: 35487465

[ref3] Al-Tawfiq J. A. Memish Z. A. (2014). Middle East respiratory syndrome coronavirus: transmission and phylogenetic evolution. Trends Microbiol. 22, 573–579. doi: 10.1016/j.tim.2014.08.001, PMID: 25178651 PMC7133228

[ref4] Bermejo-Jambrina M. Eder J. Kaptein T. M. van Hamme J. L. Helgers L. C. Vlaming K. E.. (2021). Infection and transmission of SARS-CoV-2 depend on heparan sulfate proteoglycans. EMBO J. 40:e106765. doi: 10.15252/embj.2020106765, PMID: 34510494 PMC8521309

[ref5] Bonacorsi A. Trespidi G. Scoffone V. C. Irudal S. Barbieri G. Riabova O.. (2024). Characterization of the dispirotripiperazine derivative PDSTP as an antibiotic adjuvant and antivirulence compound against *Pseudomonas aeruginosa*. Front. Microbiol. 15, 1357–1708. doi: 10.3389/fmicb.2024.1357708, PMID: 38435690 PMC10904629

[ref6] Cagno V. Tseligka E. D. Jones S. T. Tapparel C. (2019). Heparan sulfate proteoglycans and viral attachment: true receptors or adaptation bias? Viruses 11:596. doi: 10.3390/v11070596, PMID: 31266258 PMC6669472

[ref7] Center for Disease Control and Prevention. (2023) “COVID-19 Data Review”. Available at: www.cdc.gov/coronavirus/2019-ncov/science/data-review/ (accessed September 5, 2023).

[ref8] Chang C. W. Leu Y. L. Horng J. T. (2012). Daphne Genkwa sieb. Et zucc. water-soluble extracts act on enterovirus 71 by inhibiting viral entry. Viruses 4, 539–556. doi: 10.3390/v4040539, PMID: 22590685 PMC3347322

[ref9] Chen Y. Liu Q. Guo D. (2020). Emerging coronaviruses: genome structure, replication, and pathogenesis. J. Med. Virol. 92, 418–423. doi: 10.1002/jmv.25681, PMID: 31967327 PMC7167049

[ref10] Chhabra M. Doherty G. G. See N. W. Gandhi N. S. Ferro V. (2021). From cancer to COVID-19: a perspective on targeting heparan sulfate-protein interactions. Chem. Rec. 21, 3087–3101. doi: 10.1002/tcr.202100125, PMID: 34145723 PMC8441866

[ref11] Chu H. Hu B. Huang X. Chai Y. Zhou D. Wang Y.. (2021). Host and viral determinants for efficient SARS-CoV-2 infection of the human lung. Nat. Commun. 12:134. doi: 10.1038/s41467-020-20457-w, PMID: 33420022 PMC7794309

[ref12] Clausen T. M. Sandoval D. R. Spliid C. B. Pihl J. Perrett H. R. Painter C. D.. (2020). SARS-CoV-2 infection depends on cellular heparan sulfate and ACE2. Cell 183, 1043–1057.e15. doi: 10.1016/j.cell.2020.09.033, PMID: 32970989 PMC7489987

[ref13] Daelemans D. Pauwels R. De Clercq E. Pannecouque C. (2011). A time-of-drug addition approach to target identification of antiviral compounds. Nat. Protoc. 6, 925–933. doi: 10.1038/nprot.2011.330, PMID: 21637207 PMC7086561

[ref14] De Pasquale V. Quiccione M. S. Tafuri S. Avallone L. Pavone L. M. (2021). Heparan sulfate proteoglycans in viral infection and treatment: a special focus on SARS-CoV-2. Int. J. Mol. Sci. 22:6574. doi: 10.3390/ijms22126574, PMID: 34207476 PMC8235362

[ref15] Dohme A. Knoblauch M. Egorova A. Makarov V. Bogner E. (2022). Broad-spectrum antiviral diazadispiroalkane core molecules block attachment and cell-to-cell spread of herpesviruses. Antivir. Res. 206:105402. doi: 10.1016/j.antiviral.2022.105402, PMID: 36007600

[ref16] Egorova A. Bogner E. Novoselova E. Zorn K. M. Ekins S. Makarov V. (2021). Dispirotripiperazine-core compounds, their biological activity with a focus on broad antiviral property, and perspectives in drug design (mini-review). Eur. J. Med. Chem. 211:113014. doi: 10.1016/j.ejmech.2020.113014, PMID: 33218683 PMC7658596

[ref17] Hao W. Ma B. Li Z. Wang X. Gao X. Li Y.. (2021). Binding of the SARS-CoV-2 spike protein to glycans. Sci. Bull (Beijing). 66, 1205–1214. doi: 10.1016/j.scib.2021.01.010, PMID: 33495714 PMC7816574

[ref18] Harcourt J. Tamin A. Lu X. Kamili S. Sakthivel S. K. Murray J.. (2020). Severe acute respiratory syndrome coronavirus 2 from patient with coronavirus disease, United States. Emerg. Infect. Dis. 26, 1266–1273. doi: 10.3201/eid2606.200516, PMID: 32160149 PMC7258473

[ref19] Hayashida K. Aquino R. S. Park P. W. (2022). Coreceptor functions of cell surface heparan sulfate proteoglycans. Am. J. Physiol. Cell Physiol. 322, C896–C912. doi: 10.1152/ajpcell.00050.2022, PMID: 35319900 PMC9109798

[ref20] Hoffmann M. Kleine-Weber H. Schroeder S. Krüger N. Herrler T. Erichsen S.. (2020). SARS-CoV-2 cell entry depends on ACE2 and TMPRSS2 and is blocked by a clinically proven protease inhibitor. Cell 181, 271–280.e8. doi: 10.1016/j.cell.2020.02.052, PMID: 32142651 PMC7102627

[ref21] Hu Y. Jo H. DeGrado W. F. Wang J. (2022). Brilacidin, a COVID-19 drug candidate, demonstrates broad-spectrum antiviral activity against human coronaviruses OC43, 229E, and NL63 through targeting both the virus and the host cell. J. Med. Virol. 94, 2188–2200. doi: 10.1002/jmv.27616, PMID: 35080027 PMC8930451

[ref22] Hu Y. Meng X. Zhang F. Xiang Y. Wang J. (2021). The in vitro antiviral activity of lactoferrin against common human coronaviruses and SARS-CoV-2 is mediated by targeting the heparan sulfate co-receptor. Emerg. Microbes Infect. 10, 317–330. doi: 10.1080/22221751.2021.1888660, PMID: 33560940 PMC7919907

[ref23] Kim S. Y. Jin W. Sood A. Montgomery D. W. Grant O. C. Fuster M. M.. (2020). Characterization of heparin and severe acute respiratory syndrome-related coronavirus 2 (SARS-CoV-2) spike glycoprotein binding interactions. Antivir. Res. 181:104873. doi: 10.1016/j.antiviral.2020.104873, PMID: 32653452 PMC7347485

[ref24] Klimstra W. B. Tilston-Lunel N. L. Nambulli S. Boslett J. McMillen C. M. Gilliland T.. (2020). SARS-CoV-2 growth, furin-cleavage-site adaptation and neutralization using serum from acutely infected hospitalized COVID-19 patients. J. Gen. Virol. 101, 1156–1169. doi: 10.1099/jgv.0.001481, PMID: 32821033 PMC7879561

[ref25] Koehler M. Delguste M. Sieben C. Gillet L. Alsteens D. (2020). Initial step of virus entry: virion binding to cell-surface glycans. Annu. Rev. Virol. 7, 143–165. doi: 10.1146/annurev-virology-122019-070025, PMID: 32396772

[ref26] Lang J. Yang N. Deng J. Liu K. Yang P. Zhang G.. (2011). Inhibition of SARS pseudovirus cell entry by lactoferrin binding to heparan sulfate proteoglycans. PLoS One 6:e23710. doi: 10.1371/journal.pone.0023710, PMID: 21887302 PMC3161750

[ref27] Letko M. Marzi A. Munster V. (2020). Functional assessment of cell entry and receptor usage for SARS-CoV-2 and other lineage B betacoronaviruses. Nat. Microbiol. 5, 562–569. doi: 10.1038/s41564-020-0688-y, PMID: 32094589 PMC7095430

[ref28] Liu L. Chopra P. Li X. Bouwman K. M. Tompkins S. M. Wolfert M. A.. (2021). Heparan sulfate proteoglycans as attachment factor for SARS-CoV-2. ACS Cent. Sci. 7, 1009–1018. doi: 10.1021/acscentsci.1c00010, PMID: 34235261 PMC8227597

[ref29] Liu J. Thorp S. C. (2002). Cell surface heparan sulfate and its roles in assisting viral infections. Med. Res. Rev. 22, 1–25. doi: 10.1002/med.1026 11746174

[ref30] Markov P. V. Ghafari M. Beer M. Lythgoe K. Simmonds P. Stilianakis N. I.. (2023). The evolution of SARS-CoV-2. Nat. Rev. Microbiol. 21, 361–379. doi: 10.1038/s41579-023-00878-2 37020110

[ref31] Matsuyama S. Nao N. Shirato K. Kawase M. Saito S. Takayama I.. (2020). Enhanced isolation of SARS-CoV-2 by TMPRSS2-expressing cells. Proc. Natl. Acad. Sci. USA 117, 7001–7003. doi: 10.1073/pnas.2002589117, PMID: 32165541 PMC7132130

[ref32] Mautner L. Hoyos M. Dangel A. Berger C. Ehrhardt A. Baiker A. (2022). Replication kinetics and infectivity of SARS-CoV-2 variants of concern in common cell culture models. Virol. J. 19:76. doi: 10.1186/s12985-022-01802-5, PMID: 35473640 PMC9038516

[ref33] Milewska A. Zarebski M. Nowak P. Stozek K. Potempa J. Pyrc K. (2014). Human coronavirus NL63 utilizes heparan sulfate proteoglycans for attachment to target cells. J. Virol. 88, 13221–13230. doi: 10.1128/JVI.02078-14, PMID: 25187545 PMC4249106

[ref34] Muñoz-Fontela C. Dowling W. E. SGP F. Gsell P. S. Riveros-Balta A. X. Albrecht R. A.. (2020). Animal models for COVID-19. Nature 586, 509–515. doi: 10.1038/s41586-020-2787-6, PMID: 32967005 PMC8136862

[ref35] Mycroft-West C. J. Su D. Pagani I. Rudd T. R. Elli S. Gandhi N. S.. (2020). Heparin inhibits cellular invasion by SARS-CoV-2: structural dependence of the interaction of the spike S1 receptor-binding domain with heparin. Thromb. Haemost. 120, 1700–1715. doi: 10.1055/s-0040-1721319, PMID: 33368089 PMC7869224

[ref36] Paeschke R. Woskobojnik I. Makarov V. Schmidtke M. Bogner E. (2014). DSTP-27 prevents entry of human cytomegalovirus. Antimicrob. Agents Chemother. 58, 1963–1971. doi: 10.1128/AAC.01964-13, PMID: 24419339 PMC4023779

[ref37] Paiardi G. Richter S. Oreste P. Urbinati C. Rusnati M. Wade R. C. (2022). The binding of heparin to spike glycoprotein inhibits SARS-CoV-2 infection by three mechanisms. J. Biol. Chem. 298:101507. doi: 10.1016/j.jbc.2021.101507, PMID: 34929169 PMC8683219

[ref38] Perlman S. Netland J. (2009). Coronaviruses post-SARS: update on replication and pathogenesis. Nat. Rev. Microbiol. 7, 439–450. doi: 10.1038/nrmicro2147, PMID: 19430490 PMC2830095

[ref39] Reed L. J. Muench H. (1938). A simple method of estimating fifty percent endpoints. Am. J. Epidemiol. 27, 493–497. doi: 10.1093/oxfordjournals.aje.a118408

[ref40] Sanna G. Madeddu S. Murgia G. Serreli G. Begala M. Caboni P.. (2020). Potent and selective activity against human immunodeficiency virus 1 (HIV-1) of Thymelaea hirsuta extracts. Viruses 12:664. doi: 10.3390/v12060664, PMID: 32575585 PMC7354558

[ref41] Schmidtke M. Karger A. Meerbach A. Egerer R. Stelzner A. Makarov V. (2003). Binding of a N,N'-bisheteryl derivative of dispirotripiperazine to heparan sulfate residues on the cell surface specifically prevents infection of viruses from different families. Virology 311, 134–143. doi: 10.1016/s0042-6822(03)00166-1 12832211

[ref42] Schmidtke M. Riabova O. Dahse H. M. Stelzner A. Makarov V. (2002). Synthesis, cytotoxicity and antiviral activity of N,N'-bis-5-nitropyrimidyl derivatives of dispirotripiperazine. Antivir. Res. 55, 117–127. doi: 10.1016/s0166-3542(02)00014-1 12076756

[ref43] Selinka H. C. Florin L. Patel H. D. Freitag K. Schmidtke M. Makarov V. A.. (2007). Inhibition of transfer to secondary receptors by heparan sulfate-binding drug or antibody induces noninfectious uptake of human papillomavirus. J. Virol. 81, 10970–10980. doi: 10.1128/JVI.00998-07, PMID: 17686860 PMC2045555

[ref44] Sidebottom D. B. Smith D. D. Gill D. (2021). Safety and efficacy of antivirals against SARS-CoV-2. BMJ 375:n2611. doi: 10.1136/bmj.n2611, PMID: 34711614

[ref45] Tandon R. Sharp J. S. Zhang F. Pomin V. H. Ashpole N. M. Mitra D.. (2021). Effective inhibition of SARS-CoV-2 entry by heparin and enoxaparin derivatives. J. Virol. 95, e01987–e01920. doi: 10.1128/JVI.01987-20, PMID: 33173010 PMC7925120

[ref46] U.S. Food & Drug Administration. (2023). “Coronairus (COVID-19) | Drugs”. Available at: www.fda.gov/drugs/emergency-preparedness-drugs/coronavirus-covid-19-drugs (accessed September 5, 2023).

[ref47] World Health Organization. (2023). “Coronavirus disease (COVID-19)”. Available at: www.who.int/health-topics/coronavirus (accessed September 5, 2023).

[ref48] Zhang Q. Chen C. Z. Swaroop M. Xu M. Wang L. Lee J.. (2020). Heparan sulfate assists SARS-CoV-2 in cell entry and can be targeted by approved drugs in vitro. Cell Discov. 6:80. doi: 10.1038/s41421-020-00222-5, PMID: 33298900 PMC7610239

[ref49] Zumla A. Chan J. F. Azhar E. I. Hui D. S. Yuen K. Y. (2016). Coronaviruses - drug discovery and therapeutic options. Nat. Rev. Drug Discov. 15, 327–347. doi: 10.1038/nrd.2015.37, PMID: 26868298 PMC7097181

