# Supplementary material for: Efficacy of dispirotripiperazine PDSTP in a golden Syrian hamster model of SARS-CoV-2 infection
Source: Front Microbiol. 2025 Mar 10;16:1546946. doi: 10.3389/fmicb.2025.1546946 (PMC11931052; doi:10.3389/fmicb.2025.1546946)
Supplement: Supplementary file 1 [file Data_Sheet_1.pdf]

Histologic study of lungs of animals infected with SARS CoV-2 virus on the background of PDSTP application at different doses

Histological study of lungs of intact animals

Golden Syrian hamsters anesthetized with zoletil-xylazine were removed from the experiment after 3 days by exsanguination of the organism by transection of the inferior vena cava. This euthanasia procedure affected the state of the lung tissue of the animals. Uneven airiness of the parenchyma with the appearance of multiple small foci of microatelectasis combined with centriacinar and insignificant amount of panacinar emphysemas diffusely distributed over the whole area of the lobes was noted in them. Single erythrocytes and plasma were detected in the lumen of alveoli, wall localization without infiltrate, as well as small foci of hemorrhages, which was taken into account in further analysis of microdrugs of other groups. In blood vessels of different profiles we observed manifestations of muscular tissue dystonia, from expansion of their lumen to complete closure with wall recession in some areas, slowing of blood flow with uneven distribution of plasma and blood form elements. Disturbance of muscle tone was also noted for bronchial muscles. These changes were characteristic for all experimental animals and, therefore, were not taken into account when describing lung pathology. The epithelial lining of the bronchi of intact animals was characterized by a small degree of desquamation of the epithelium, and mainly forms of cells with lytic nuclei (natural aging). A moderate amount of mucous secretion of wall localization was observed (Figure 1S).

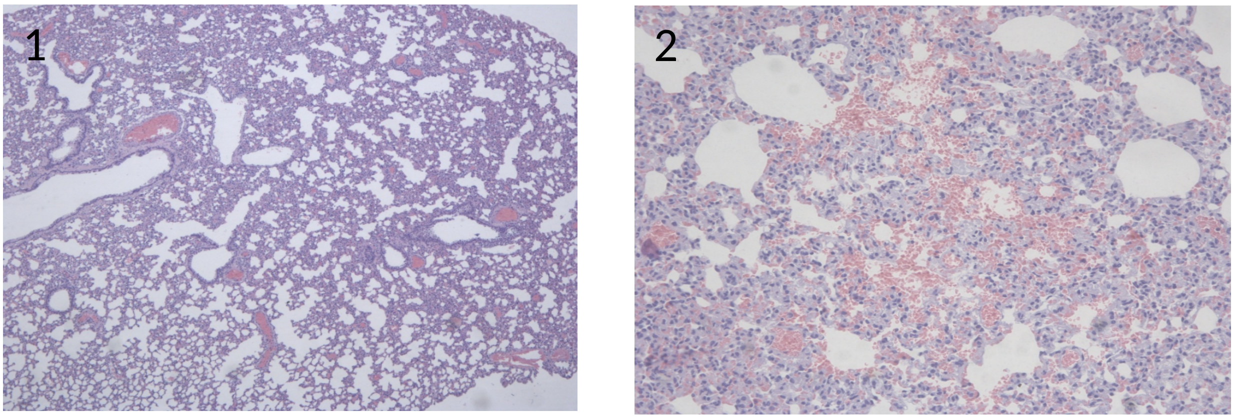

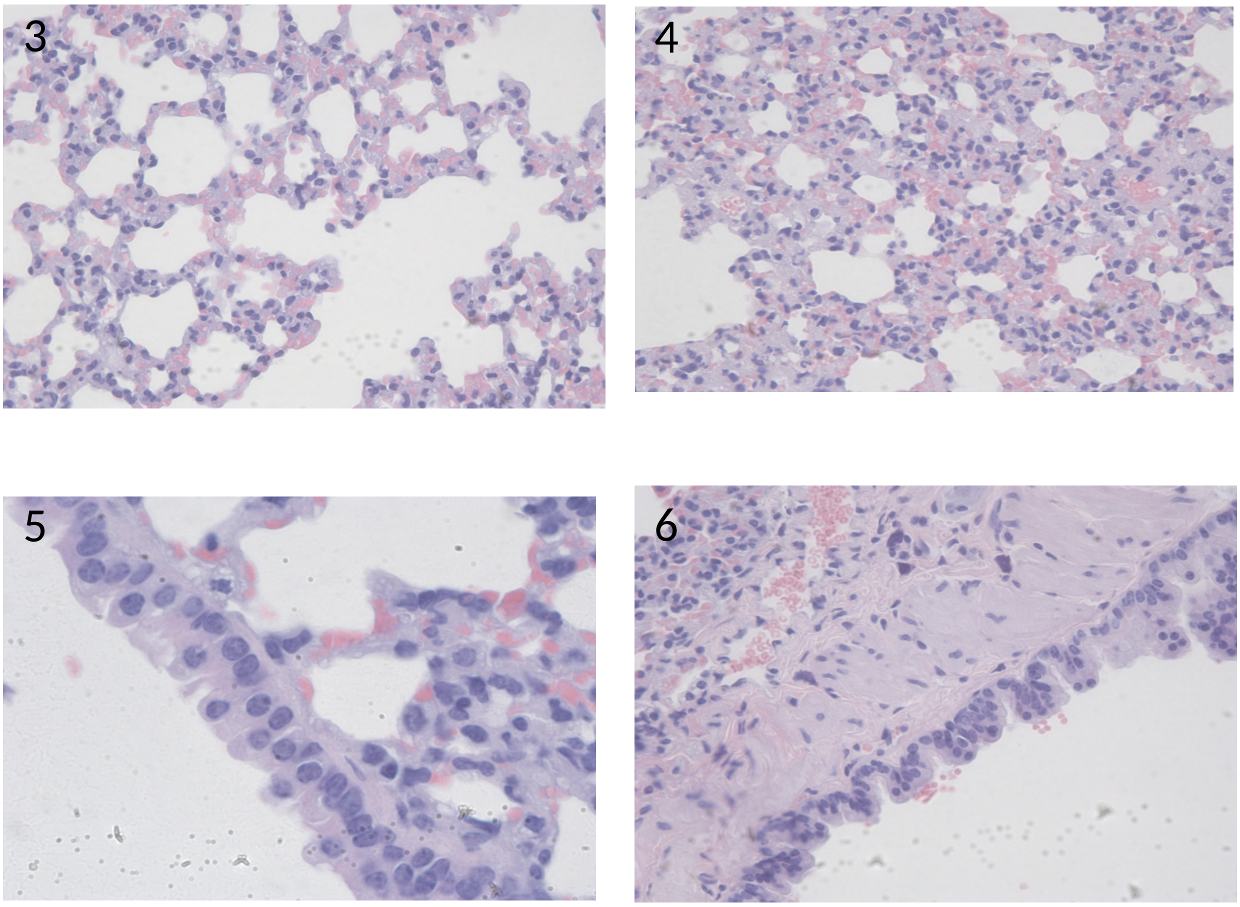

Figure 1S - Lungs of Syrian hamsters. Intact group.

1 - Combination of areas of microatelectasis, areas of normal airflow and emphysema in the tissue of the left lobe of the Syrian hamster lung after euthanasia with zoletil-xylazine anesthesia. ×10 objective. 2 - Focus of small hemorrhage. Non-large panacinar emphysema is visualized. Lens ×20. 3 - Acinar area of normal airiness. Single erythrocytes of walled intra-alveolar localization. Lens ×40. 4 - Area combining microatelectasis and moderately thickened alveolar septa. Diapedesic erythrocytes in the lumen of the alveoli. Lens ×40. 5 - A section of a small-caliber bronchus. Double row arrangement of epithelium lining. Lens ×100. 6 - A section of the wall of a large bronchus. Single walled erythrocytes. Absence of infiltration of the muscular and axillary layers. Lens ×40. Carazzi hematoxylin and eosin staining.

Histologic study of lungs of control animals on 3 days after infection with SARSCoV-2 virus

After 3 days in hamsters of the control group (infection + placebo) the state of lung tissue in general corresponded to the initial stage of the exudative phase of acute inflammation. At the same time, the airiness of the lung tissue, the area occupied by microatelectasis foci remained comparable with intact animals, however, some larger compacted airless areas were observed in the majority of animals (Figure 2S). In contrast to intact animals, a significantly larger amount of leukocytic

infiltrate and activated forms of mononuclear cells localized in alveolar septa, peribronchial interstitium, especially in airless areas was visualized. Among the cells of the whole infiltrate lytic forms or cells with karyorrhexis manifestation were detected (Figure 16). Free and groups of diapedesis erythrocytes were detected in most of the alveoli. Moderate interstitial edema of alveolar septa, edema of separate alveoli were observed in the foci of microatelectasis, in which along with active large foamy macrophages, often with lysed nuclei, segmented leukocytes in different functional state, including those phagocytized by macrophages, were observed. Separate areas of septa with lysis of nuclear elements of wall cells were noted. The bronchial lumen had free patency in general. In contrast to intact animals, in many profiles there were observed wall accumulations of desquamated lining cells, frothy macrophages, leukocytes and their detritus, free erythrocytes, increased number of secretion lumps. In relation to intact animals, the processes of epithelial desquamation were enhanced, especially in the profiles of larger bronchi. While the general integrity of the bronchial lining was preserved, small foci of thinning of the lining with sparse arrangement of cells, foci of dystrophically changed epitheliocytes with large and small vacuolated cytoplasm, lytic or pycnomorphic nuclei were detected in separate sections, more often in larger bronchi (Figure 3S). Penetration of bronchial lining by leukocytic cells was frequently observed, including in the form of nuclear detritus in epitheliocytes or in the bronchial lumen (Figure 3S). In the lumen of blood vessels there was increased content of blood nuclear elements, which in many profiles were in a state of adhesion on the endothelium, in some areas penetrating their walls with moderate accumulation in the adventitia (Figure 4S).

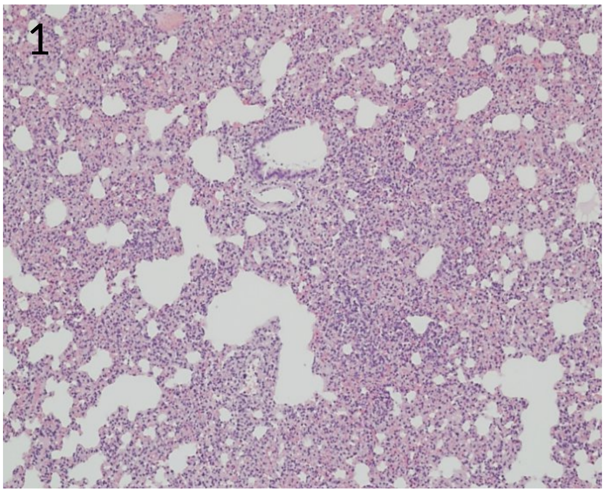

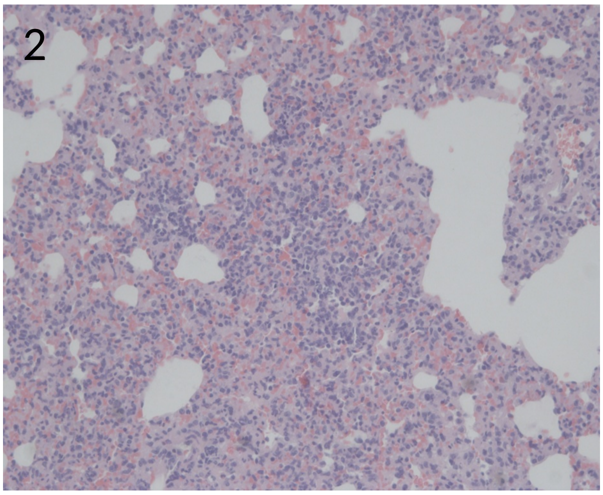

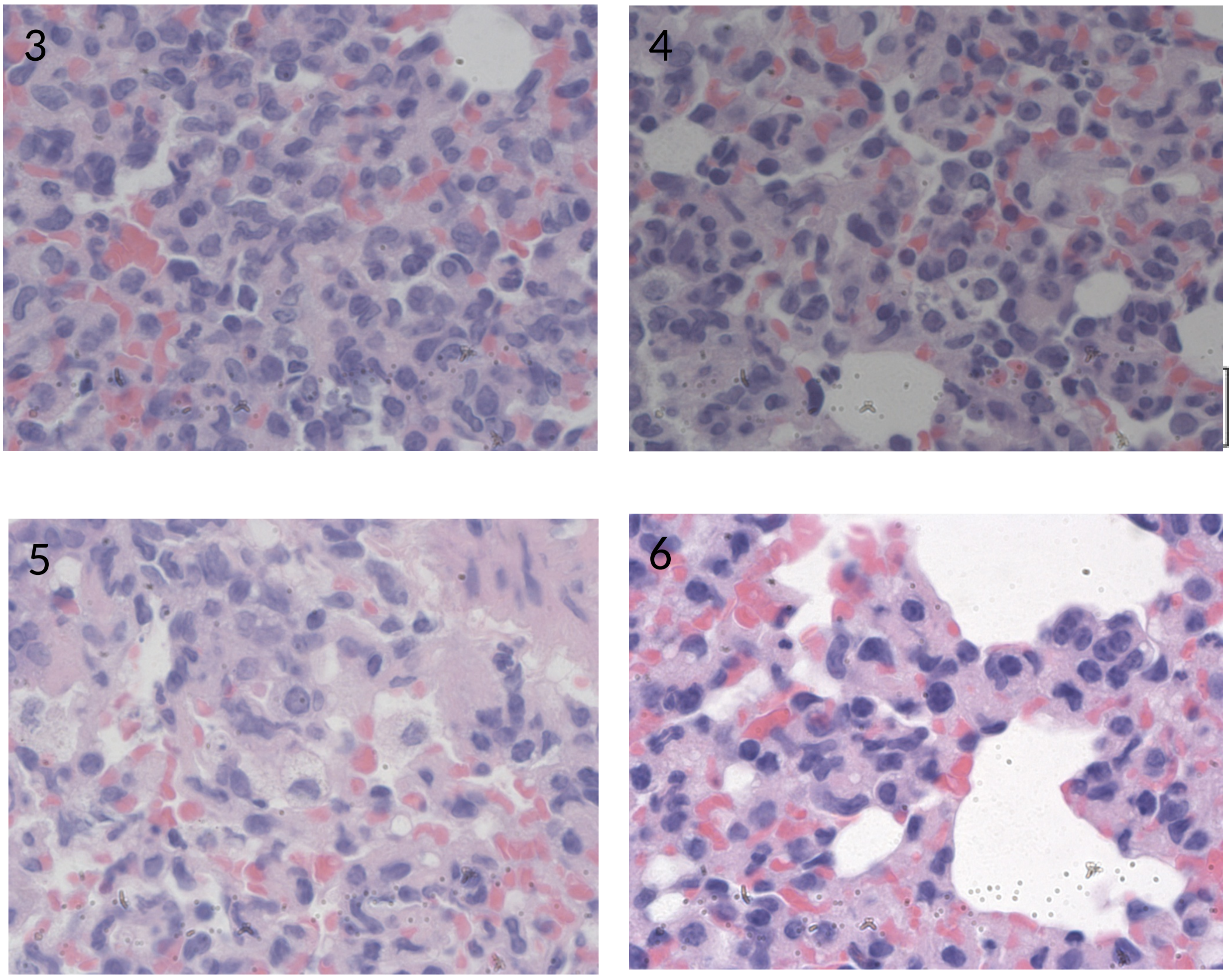

Figure 2S - Lungs of Syrian hamsters of the control group 3 days after infection

1 - Uneven airiness of animal lung tissue 3 days after infection. Lens ×4. 2 - Large focus of atelectasis with infiltrate. Lens ×20. 3 - The same area. High magnification. Leukocytic-monocytic infiltrate in edematous septa. Lens ×100. 4 - Acinar section. Active forms of macrophages in the lumen of alveoli with leukocytic detritus. Lens ×100. 5 - Acinar area with development of alveolar edema and frothy macrophages in the lumen. Lens ×100. 6 - Increased leukocytic-monocytic infiltrate in the alveolar septa. Lens ×100. Hematoxylin and eosin staining.

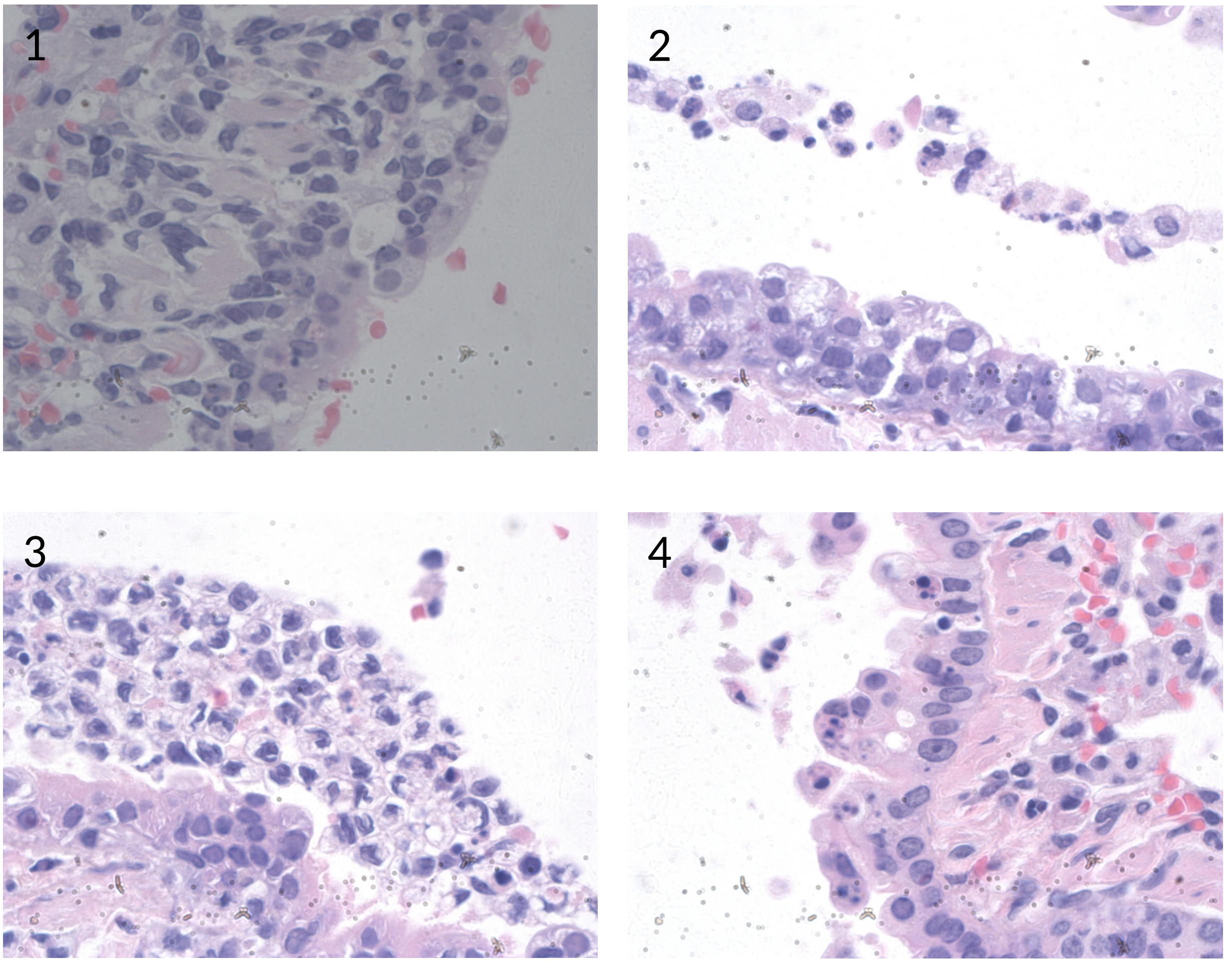

Figure 3S - Fragments of the walls of large bronchi of Syrian hamsters of the control group 3 days after infection

1 - Inflammatory infiltrate in the muscularis, intrinsic lamina, and epithelial lining of a large bronchus. Lens ×100. 2 - Vacuolated dystrophy of epitheliocytes. Accumulation of inflammatory infiltrate in the lumen of the bronchus. Lens ×100. 3 - In the lumen of the bronchus leukocytic detritus, macrophages, dead epithelial cells. Lens ×100. 4 - Leukocytic detritus in the epithelial layer. Lens ×100.

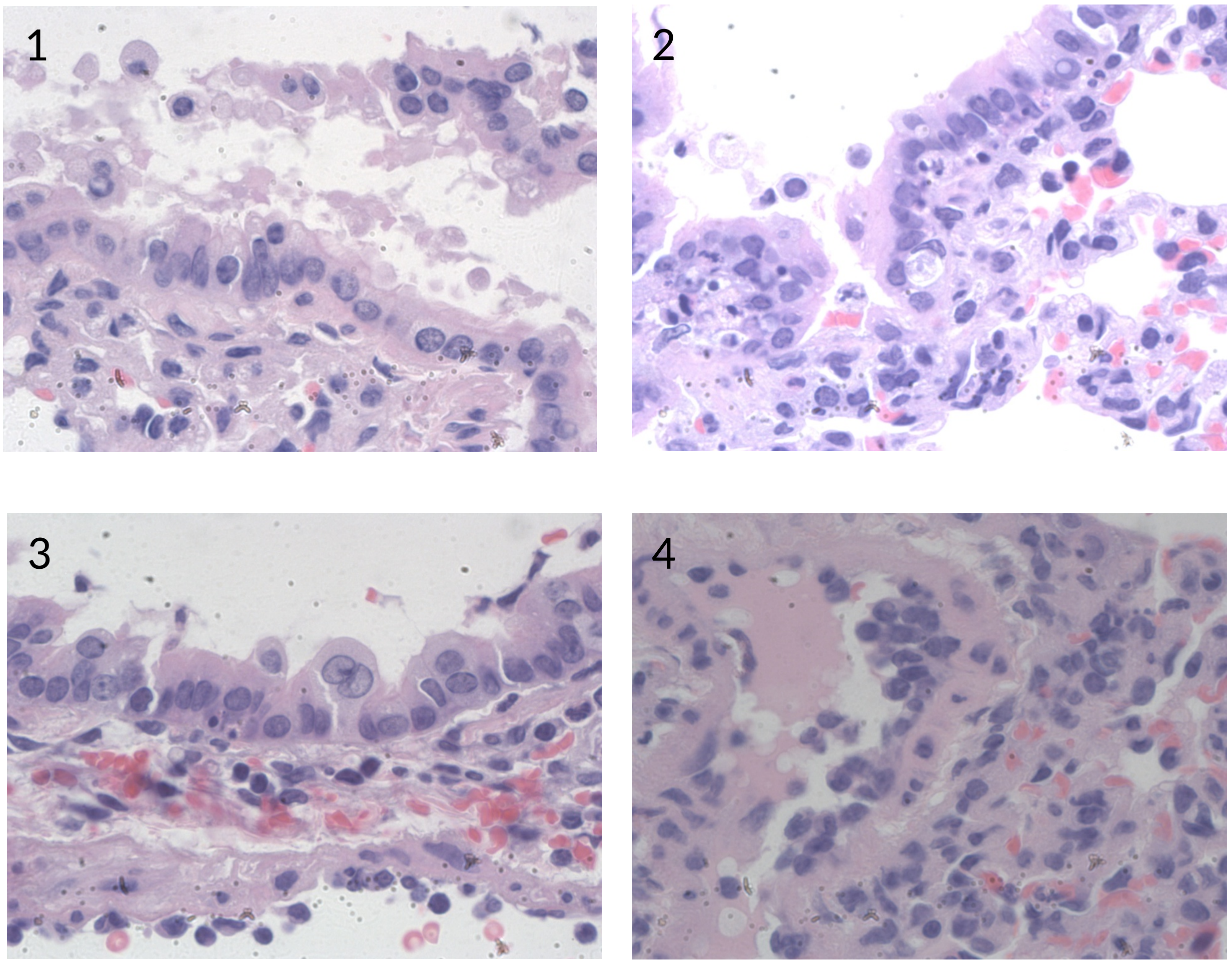

Figure 4S - Fragments of small bronchial walls of Syrian hamsters of the control group 3 days after infection

1 - Lumps of secretion, desquamated bronchial epithelium in the lumen. Lens ×100. 2 - Terminal bronchiole. Leukocytes, macrophages with detritus in the epithelial layer of the bronchial lining. Lens ×100. 3 - Fragment of a small bronchus, pronounced capillary hemorrhage, diapedesis of erythrocytes in its own lamina. Lens ×100. 4 - Adhesion of blood nuclear elements on the endothelium of the vessel. Infiltrate accumulation in the intermuscular space. Lens ×100.

Histologic study of lungs of animals infected with SARS-CoV-2 virus on the background of PDSTP application, 3 days after infection

In the majority of animals receiving PDSTP at a dose of 10 mg/kg, 3 days after infection, the degree and uniformity of lung parenchyma airiness was higher than in animals of the control group, which was determined by incomplete recession of alveolar septa with the presence of multiple zones of microatelectasis (Figure 5S). The degree of interstitial edema of alveolar septa was moderate and had a focal character comparable to control animals, whereas the saturation of septa with cells of inflammatory infiltrate was lower throughout the parenchyma. Segmented leukocytes in the majority of animals were found in smaller numbers, practically within the capillary wall or septum stroma, including manifestations of karyolysis. Mononuclei were

dominant. Macrophage cells with manifestations of pronounced phagocytic activity (vacuolized, large in size) were detected in a single number. In contrast to control animals, the patterns of erythrocyte diapedesis of wall localization were visualized in a smaller volume. Two animals out of six had small foci of erythrocyte accumulations with manifestations of wall alveolar edema in one of them. Leukocytes in the lumen of alveoli were not determined. In the lumen of vessels the content of blood nuclear elements was insignificant at the level of intact animals, without their adhesion on the endothelium. In contrast to control animals, there were no pictures of vacuolar dystrophy of bronchial epithelium (both large and small), focal necrosis of epithelium and its infiltration with leukocytes. The process of desquamation of epitheliocytes, the content of secretion granules remained at the level of intact animals. In the bronchial lumen there were no accumulations of activated forms of macrophages, leukocytes together with sloughed epitheliocytes. In one of the animals of the group moderate manifestations of vasculitis and presence of foci of infiltration of muscles of a large bronchus were noted.

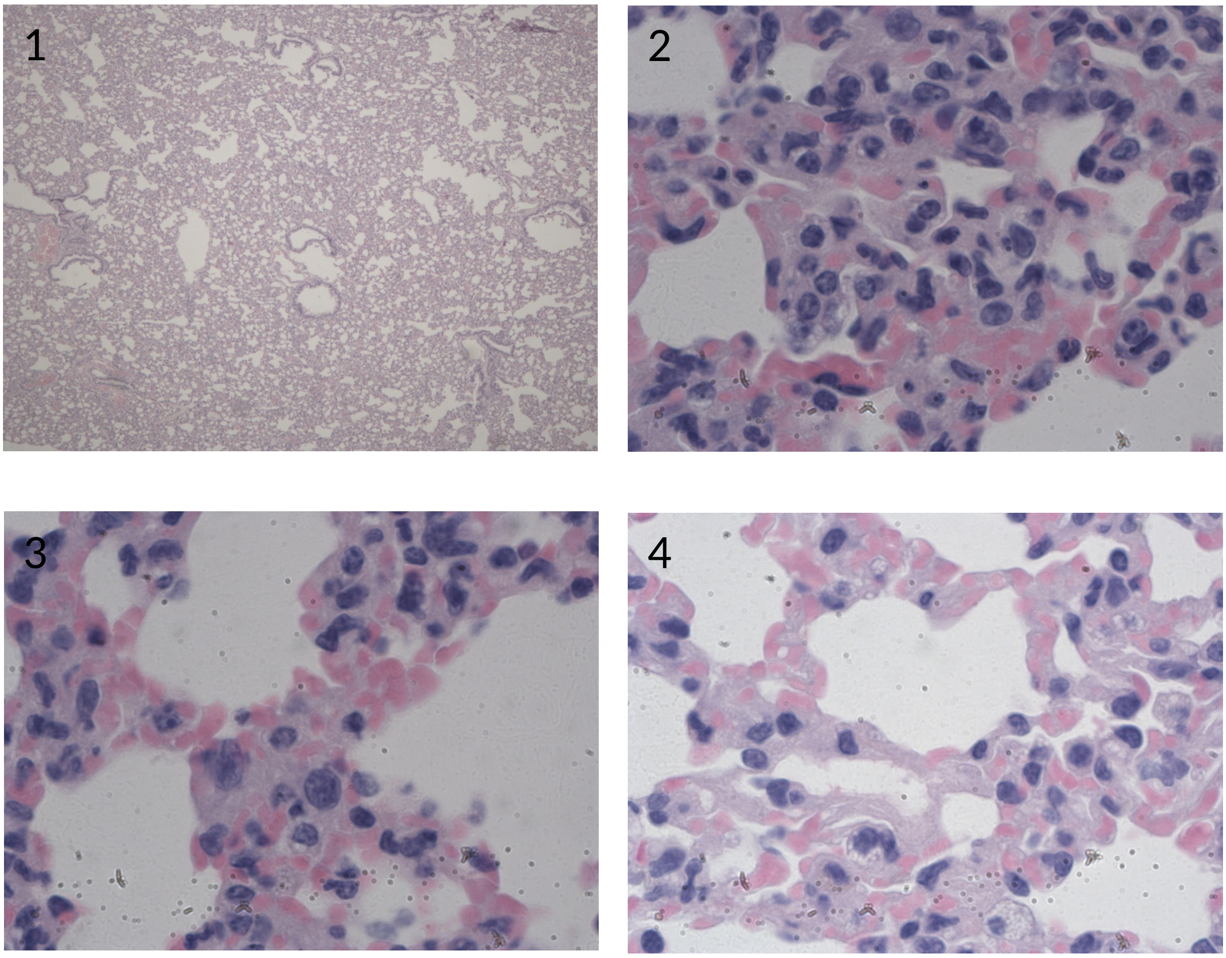

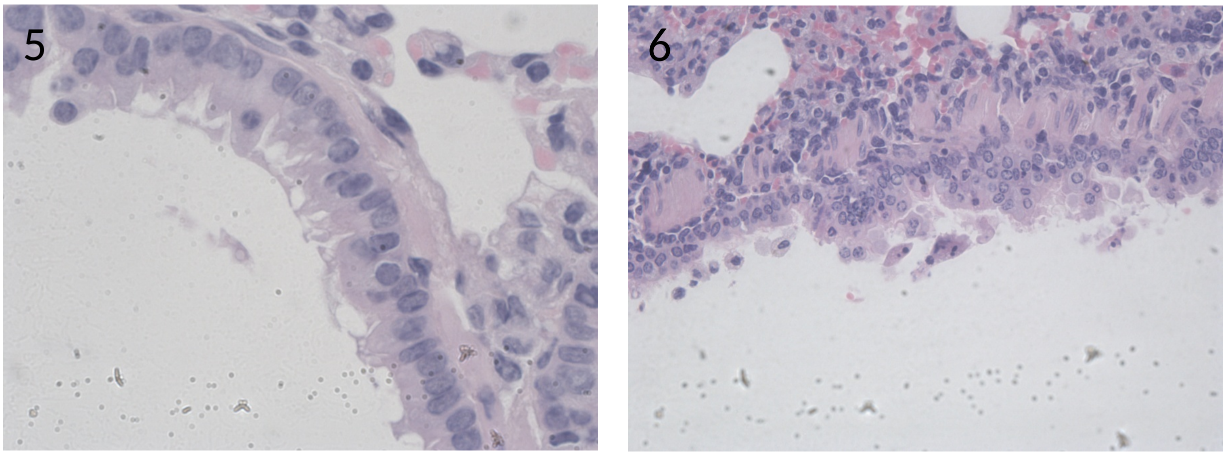

Figure 5S - Lung fragments of Syrian hamsters of the group administered PDSTP at a dose of 10 mg/kg 3 days after infection

1 - Uniform airiness of lung parenchyma without foci of expressed tissue thickening. Lens ×4. 2 - Moderate mononuclear infiltration of the stroma of alveolar septa. Lens ×100. 3 - Diapedosed erythrocytes in the lumen of alveoli. Lytic forms of leukocytes are seen in septa with moderate infiltrate. Lens ×100. 4 - Focus of denudation of alveolar septa. Moderate amount of infiltrate, predominantly round cellular. Lens ×40. 5 - Moderate amount of secretion on the surface of the bronchial lining of a small-caliber bronchus. No visualization of infiltrate and desquamated epitheliocytes. Lens ×100. 6 - Fragment of the lining of a large bronchus. In the lumen frothy macrophages, leukocytic detritus. Inflammatory infiltrate in the muscular layer. Lens ×40. Hematoxylin and eosin staining.

When the dose of PDSTP was increased to 20 mg/kg, the parenchyma airiness remained practically unchanged compared to the animals treated with a lower dose (Figure 6S). Multiple microatelectasis diffusely distributed throughout the parenchyma in combination with emphysematous foci and areas of normal airiness were detected. The septal cell saturation of inflammatory infiltrate was also comparable with the 10 mg/kg dose of the drug and reduced relative to the control. In most of the studied parameters (presence of dystonia of muscular tissue of vessels and bronchi, visualization of interstitial and intra-alveolar edema, tissue distribution and composition of inflammatory infiltrate) no special difference was found between the two doses of the drug, except for one animal, in which dominance of leukocyte content was noted in the inflammatory infiltrate of interstitium of alveolar septa. Differences between the groups of treated animals were manifested in more pronounced changes in the state of airway walls. At the drug dose of 20 mg/kg, increased desquamation and increased secretory activity of epitheliocytes lining both large and small bronchi were observed in almost all animals of the group (Figure 7S). In the lumen of medium and large bronchi there were observed accumulations of inflammatory infiltrate cells mainly consisting of macrophages and leukocytic detritus. In the epithelium of the majority of large bronchi extensive foci of dystrophy, necrobiosis of cells, presence of leukocytic

detritus were determined. In a part of animals in the underlying muscle tissue and bronchial interstitium small foci of inflammatory infiltrate, mainly consisting of mononuclei, were visualized, and adhesion of blood nuclear elements was observed on the endothelium of adjacent vessels on the bronchial side, which was characteristic of most animals of the control group (infection + placebo). In some cases, the development of edema of intermuscular stroma of the bronchial wall was noted. The above mentioned changes in general corresponded to those described for control animals for this period of the study, although the volume of necrotically changed cells and the severity of vacuolar dystrophy of the epithelium of large bronchi were manifested to a lesser extent.

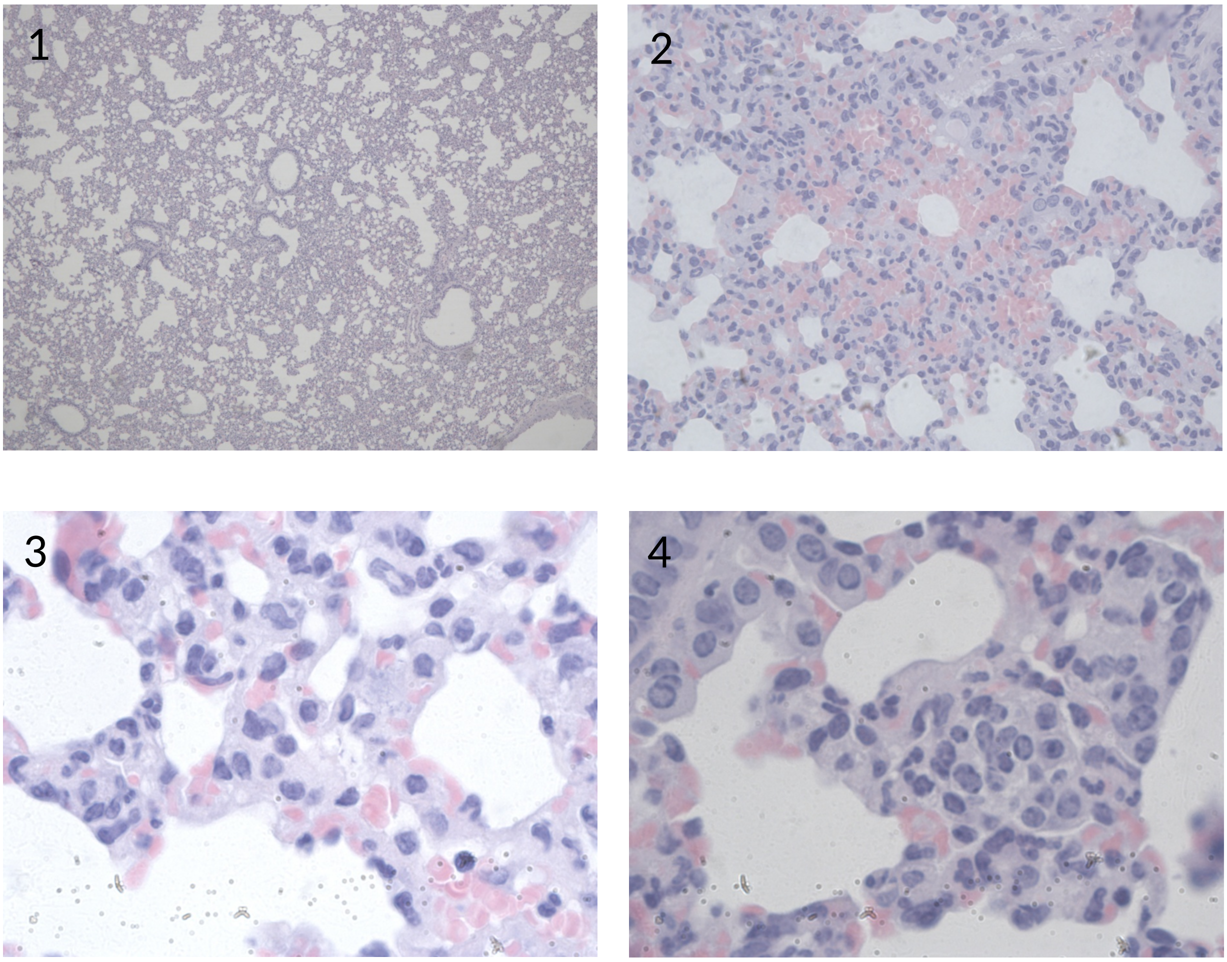

Figure 6S - Lung fragments of Syrian hamsters of the PDSTP treatment group at a dose of 20 mg/kg 3 days after infection

1 - Combination of microatelectasis, normal airflow, and emphysematous areas in the parenchyma of the left lobe of the lung. Lens ×4. 2 - Small focus of hemorrhage in acinar structures. Lens ×40. 3, 4 - Alveolar septa. Predominantly mononuclear infiltrate. Lens ×100.

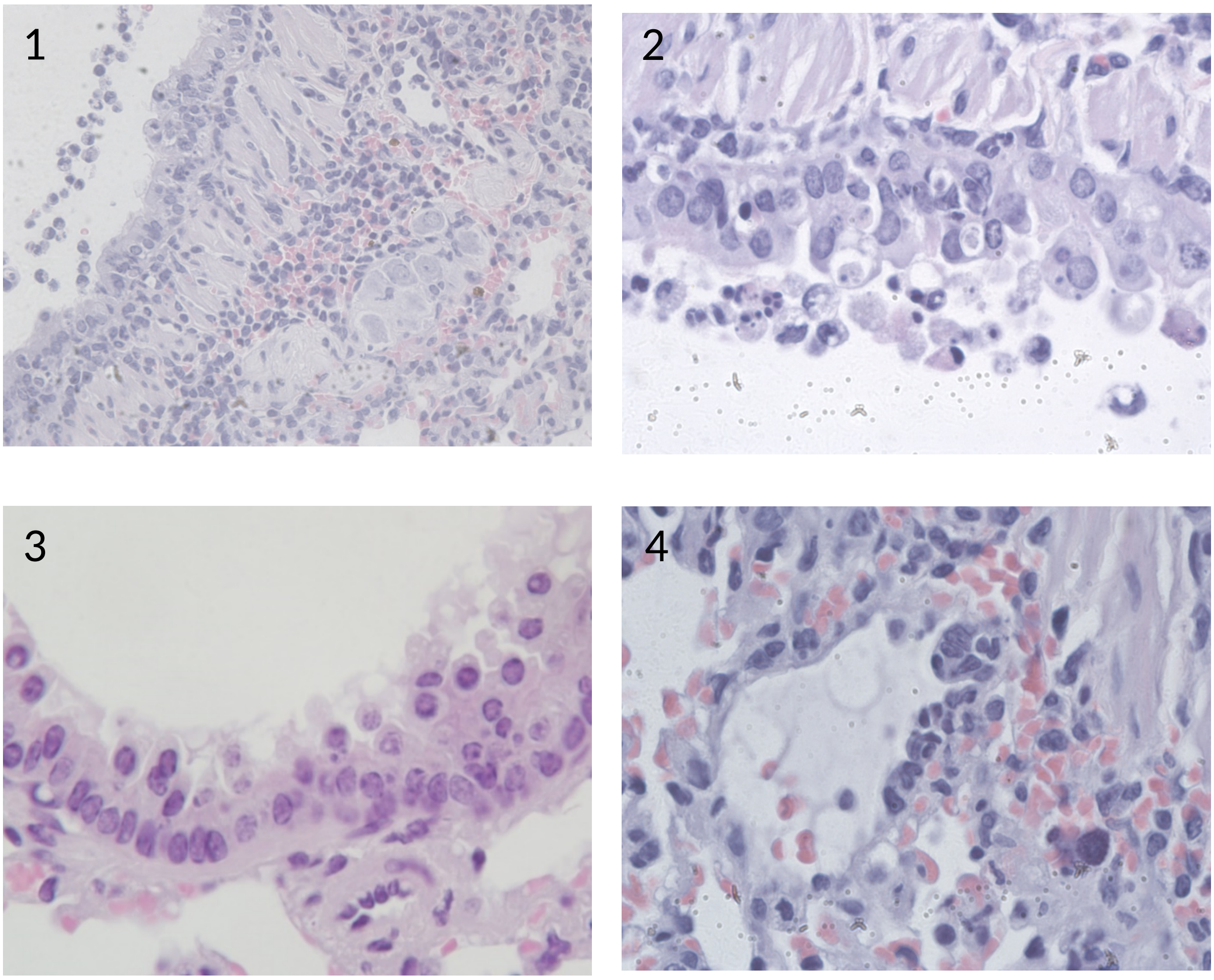

Figure 7S - Bronchial lung fragments of Syrian hamsters of the PDSTP application group at a dose of 20 mg/kg 3 days after infection

1 - Large bronchus. Hemorrhage in the peribronchial tissue. Inflammatory infiltration of all layers of bronchial lining. Noticeable amount of desquamated epithelium and cells of inflammatory infiltrate in the lumen of the bronchus. Lens ×40. 2 - Inflammatory infiltrate in the intrinsic lamina of bronchial mucosa, penetration of epithelial lining, swelling of epithelial cell nuclei, cellular detritus in the soma of macrophages. Edema of the muscular tissue of the bronchial sheath. Lens ×100. 3 - Small bronchus. Mucous secretion, normal bronchial epithelium. Lens ×100. 4 - Blood vessel with a focus of adhesion of inflammatory infiltrate cells on the endothelium. Lens ×100.

Animals receiving PDSTP at a maximum dose of 40 mg/kg also showed irregularity of tissue airiness with alternating areas of microatelectasis and emphysema. The volumes of air tissue remained at the level of animals of other groups and were not less than 50 % of the whole slice area (Figure 8S). The presence of larger emphysematous areas of panacinar type was observed in some animals. As in previous studies, in all animals of the group expressed manifestations of dystonia of muscular tissue of vessels and bronchi were detected. The presence of wall diapedesis erythrocytes in the lumen of alveoli, small single hemorrhages remained common. The condition of alveolar septa by manifestations of interstitial edema, severity of infiltration did not significantly

differ from animals with low doses of the therapeutic preparation. However, in half of the animals there was noted an increase in the frequency of observation of prestenotic alveolar edema. In relation to the previous described model, in the state of large and medium bronchi there was observed an increase of pathological manifestations such as desquamation of epitheliocytes lining, presence of multiple small foci with pycnosis of nuclei, vacuolar dystrophy of cytoplasm, lysis of separate epithelial cells, presence of significant amount of leukocytic detritus in the stratum or lumen of bronchi. The manifestations of edema of the muscular wall of bronchi and vessels were comparable to those described earlier in treated animals at a dose of 20 mg/kg.

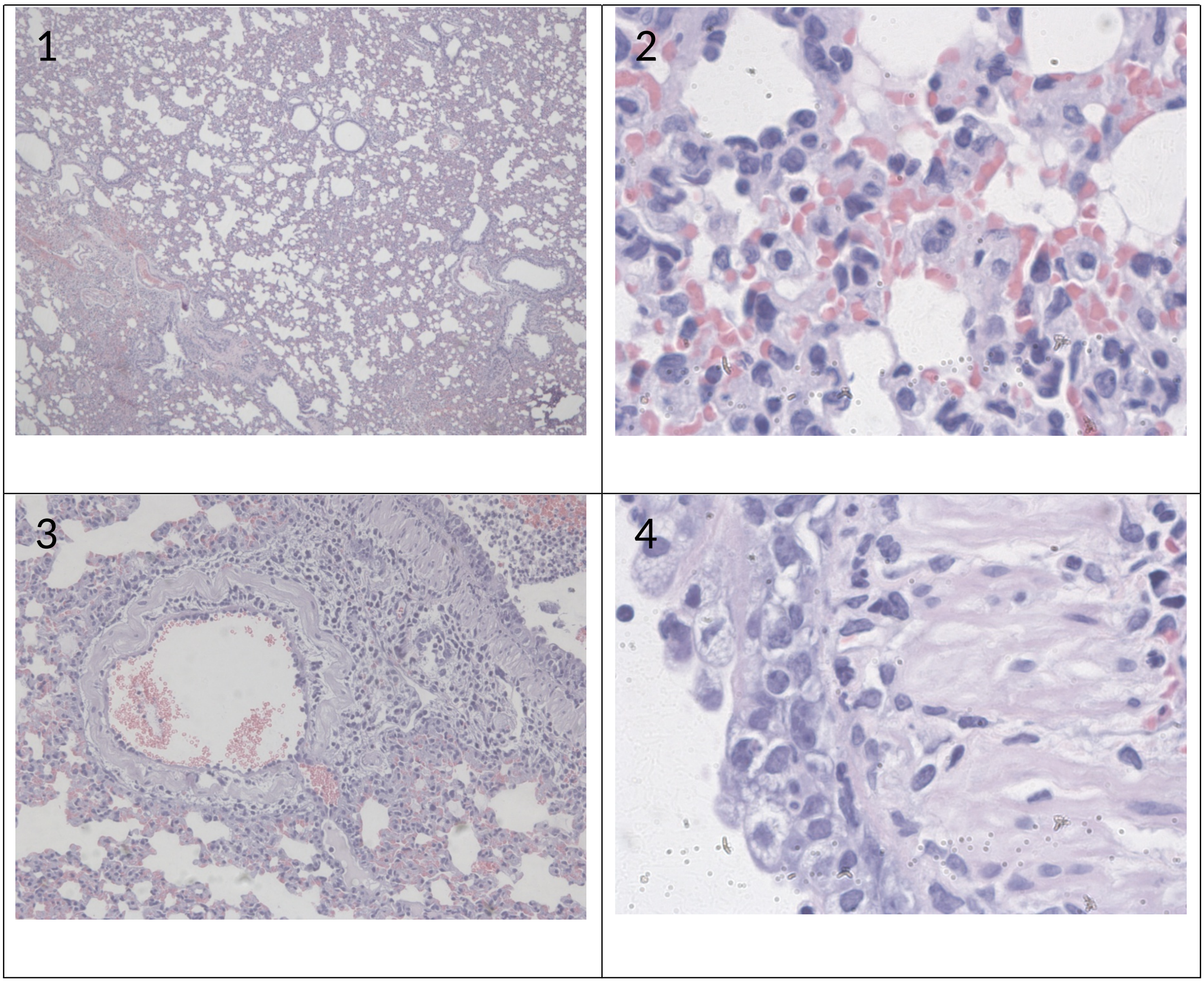

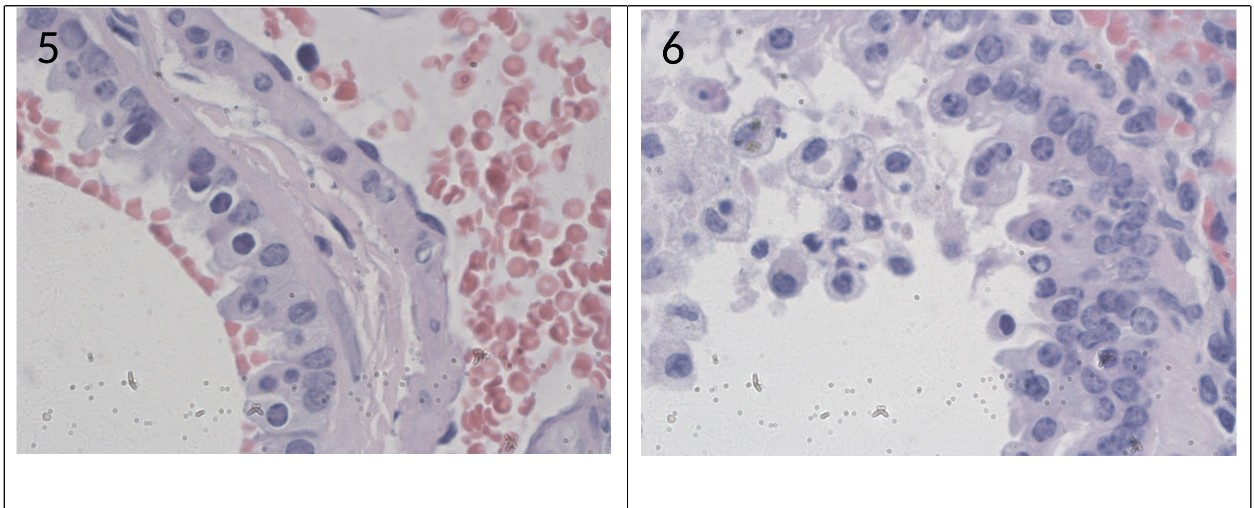

Figure 8S - Fragments of acinar structures and bronchi of lungs of Syrian hamsters of the AFS PDSTP group at a dose of 40 mg/kg 3 days after infection

A - The state of dystelectasis. Lens ×4. B -Significant amount of edematous fluid in the alveolar lumen. Moderate mononuclear infiltrate of the septa. Adhesion of blood nuclear cells on the endothelium of the vessel. Lens ×100. B - Pulmonary artery next to a large bronchus. Adhesion of blood cell elements on the walls of the vessel with penetration. Lens ×20. D - The lining of a large bronchus. Vacuolated dystrophy of epitheliocytes, infiltration of mucosa and muscular layer of bronchus. Leukocytes in the epithelium of the lining. Moderate edema of the muscular tissue. Lens ×40. D - Fragment of a small bronchus. Karyolysis of a significant number of epitheliocytes. E - Terminal bronchiole. Inflammatory infiltrate in its lumen (frothy macrophages and leukocytic detritus). Lens ×100.

Thus, the application of PDSTP in doses of 10, 20 and 40 mg/kg and GLF PDSTP in a dose of 20 mg/kg 3 days after infection with SARS-CoV-2 virus promoted greater preservation of parenchyma airiness due to the absence of denser airless areas. In all studied doses of preparations, the amount of inflammatory infiltrate in the parenchyma was reduced, among which mononuclear forms dominated (in animals without prophylaxis, segmented leukocytes prevailed). There were no significant differences in the manifestations of interstitial and alveolar edema. In relation to the control, the development of pathological changes in the structures of the airways was observed to a slightly lesser extent in all experimental groups. The summarized information is presented in Table 1S.

Table 1S - Informative micromorphologic signs of lung tissue lesions in golden Syrian hamsters at 3 days after infection against and PDSTP treatment

Characteristic morphological signs	Number of animals in the group with this trait				
	intact	infected control	PDSTP 10 мг/кг	PDSTP 20 мг/кг	PDSTP 40 мг/кг
Vascular disorders					
Annexin leukocytes	0/6	3/6	1/6	3/6	3/6
Leukocytosis	0/6	0/6	1/6	0/6	1/6
Diapedesis	6/6	6/6	6/6	6/6	6/6
Small focal hemorrhages	4/6	3/6	2/6	2/6	3/6
Medium focal hemorrhages	0/6	0/6	0/6	0/6	1/6
Vasculitis	0/6	0/6	1/6	2/6	0/6
Paravasal edema	0/6	0/6	0/6	0/6	2/6
Condition of the lung tissue					
Microatelectasis	6/6	6/6	6/6	6/6	6/6
Foci of inflammation S< 50%	0/6	4/6	0/6	0/6	1/6
Foci of inflammation S≥ 50%	0/6	0/6	0/6	0/6	0/6
Condition of the interalveolar septum					
Foci of thickening due to edema	0/6	6/6	0/6	0/6	1/6
with cell infiltration	0/6	6/6	6/6	6/6	6/6
wall-mounted	0/6	6/6	1/6	2/6	6/6
focal with cell infiltration	0/6	0/6	0/6	0/6	0/6
Condition of the bronchial epithelium					
Dystrophy	0/6	5/6	0/6	4/6	3/6
Necrosis of single cells	0/6	5/6	3/6	3/6	4/6
Leukocytes	0/6	3/6	0/6	2/6	1/6
Mixed-cell infiltrate in the lumen	0/6	5/6	0/6	5/6	2/6
Condition of peribronchial tissue					
Focal cell infiltration:					
- own plate	0/6	6/6	2/6	4/6	4/6
- muscle layer	0/6	6/6	2/6	4/6	4/6
