# Supplementary material for: Efficacy of dispirotripiperazine PDSTP in a golden Syrian hamster model of SARS-CoV-2 infection
Source: Front Microbiol. 2025 Mar 10;16:1546946. doi: 10.3389/fmicb.2025.1546946 (PMC11931052; doi:10.3389/fmicb.2025.1546946)
Supplement: Supplementary file 2 [file Data_Sheet_2.docx]

**SUPPORTING INFORMATION**

**Efficacy of dispirotripiperazine PDSTP in a golden Syrian hamster model of SARS-CoV-2 infection**

Giuseppina Sanna*†^1*^*, Olga Riabova*†^2^*, Elena Kazakova*^2^*, Alexander Lepioshkin*^2^*, Natalia Monakhova*^2^*, Alessandra Marongiu*^1^*,

Gianluigi Franci^3^, Aldo Manzin*‡^1^*, Vadim Makarov*‡^2^**

^1^Department of Biomedical Sciences, Microbiology and Virology Unit, University of Cagliari, Cittadella Universitaria, Sp. 8, 09042 Monserrato (CA), Italy

^2^Federal Research Centre “Fundamentals of Biotechnology” of the Russian Academy of Sciences (Research Centre of Biotechnology RAS), Leninsky prospect, 33, build. 2, 119071 Moscow, Russia

^3^Department of Medicine, Surgery and Dentistry "Scuola Medica Salernitana", University of Salerno, 84081 Baronissi, Italy

*†*These authors contributed equally to this work

*‡*These authors shared senior authorship

***Corresponding authors:

g.sanna@unica.it, +39(070)6754161 (GS),

makarov@inbi.ras.ru, +7(495)6603430(195) (VM)

**Methods**

**Cytotoxicity Assay**

The microtetrazolium test (MTT test) was used to study the cytotoxicity of the compounds. Vero cells were seeded in 96-well plates at an initial density of 3×10^5^ cells/mL, in Gibco Dulbecco’s Modified Eagle Medium supplemented with L-glutamine and 25 mg/L kanamycin, and 10 % fetal bovine serum (FBS) (DMEM, Thermo Fisher Scientific, Waltham, MA USA). Cell cultures were then incubated at 37 °C in a 5 % CO_2_ atmosphere, in the absence or presence of serial dilutions of test compounds. The test medium used for the cytotoxic assay as well as for the antiviral assay contained 1 % of the appropriate serum. Cell viability was determined after 72 h at 37 °C by the MTT method as previously reported [1]. The cell viability was expressed as a percentage of the control. The 50% cytotoxic concentration (CC_50_) of each compound was defined as the concentration of the compound that reduced the absorbance (OD_570_) of the treated cells to 50% when compared with that of untreated cells. CC_50_ mean values were calculated from at least three independent experiments.

**Virucidal Activity Assay**

PDSTP (20 µM) was incubated with 3×10^5^ PFU/mL of SARS-COV-2 PV10734 at either 4 and 37 °C for 2 hours. The mixture of the viral sample without a test compound was used as an untreated control. At the end of the incubation period, samples were serially diluted in media and titers were determined on Vero-76 cells at high dilutions, at which the compound was not active. Virus titers were determined by plaque assay in Vero-76 cells.

**High-Performance Liquid Chromatography Analysis of PDSTP Purity**

Purity of PDSTP was analyzed by analytical high-performance liquid chromatography on an LC-20 Prominence HPLC system (Shimadzu, Kyoto, Japan) equipped with an SPD-20A Prominence UV detector at 254 nm. Chromatographic separation was carried out on a Kromasil 60-Diol HPLC column (4.6 × 250 mm, 5 μm; Nouryon, Göteborg, Sweden) at 30 °C, sample injection volume – 20 µL. A mobile phase consisting of methanol in DMSO (90:10 v/v, A), and heptafluorobutyric acid in methanol (0.5: 95.5 v/v, B) was programmed with gradient elution at a flow rate of 1300 µL/min. Data were processed with LCsolution 1.25 rus software (Shimadzu).

**Standard RNA Preparation and qRT-PCR Standard Curve Plotting**

N-gene RNA sequence was used to generate a standard curve for RT-qPCR. This sequence was produced by in vitro transcription of the pUC57 linear plasmid template containing the cloned N-gene sequence (Innova plus, Saint Petersburg, Russia) using T7 RNA polymerase (Fig. S3). To synthesize the RNA sequence of the SARS-CoV-2 N-gene, 0.5 µg of the plasmid was linearized with an EcoRV-HF restriction enzyme (New England Biolabs, Ipswich, MA USA) at 37 °C for 16 h, then the restriction enzyme was inactivated by heating at 60 °C for 20 min. The plasmid length was verified on a 1.0% agarose gel electrophoresis using a GeneRuler 1 kb Plus DNA Ladder (Thermo Fisher Scientific). Restriction product 3050 bp long was isolated from the gel using a Cleanup S-cap kit (Evrogen, Moscow, Russia). The concentration of the isolated plasmid fragment was measured on a NanoDrop 1000 spectrophotometer (Thermo Fisher Scientific).

The transcription reaction was carried out on a plasmid template using T7 RNA polymerase. The reaction mixture (20 μl) containing 100.0 ng linearized plasmid template, 2.0 µl T7 RNA polymerase (New England Biolabs), 2.0 µl RNAPol reaction buffer (New England Biolabs), supplemented with 10 µl of each ribonucleotides (ATP, UTP, GTP, CTP), and 0.5 µl RNase recombinant ribonuclease inhibitor (1 U/µl, New England Biolabs), was incubated at 37 °C for 4 h. Plasmid DNA was removed using DNase: 1.0 µl DNase (Thermo Fisher Scientific, USA) and 4.0 µl buffer were added to the tube, the reaction volume was adjusted to 40.0 µl with of nuclease-free water. The reaction was carried out was incubated at 37 °C for 4 h.

RNA was purified using an RNeasy Mini Kit (Qiagen, Hilden, Germany). To do this, the volume of the reaction mixture was adjusted to 500.0 μl, and the mixture was applied to a column and centrifuged at 8000 g for 15 s. Then the column was washed twice with 500.0 μl of buffer RPE and the procedure was repeated. The empty column was centrifuged to remove buffer RPE at 8000 g for 30 s. RNA was washed off the column with 30.0 μl of nuclease-free water. The concentration of isolated RNA was measured on a NanoDrop 1000 spectrophotometer (Thermo Fisher Scientific).

Serial 10-fold dilutions from 10^6^ to 10^1^ copies were prepared, and the number of RNA copies was calculated to plot the standard curve (Fig. S4) using the formula:

$$RNA copy number=\frac{DNA amount (ng)\cdot6.022\cdot{10}^{23}}{RNA lenght (bp)\cdot1\cdot{10}^{9}\cdot320}$$

**Figures**

**Figure S1. Virucidal activity of PDSTP**. The compound lacks virucidal activity against SARS-CoV-2 at either 4 or 37 °C.


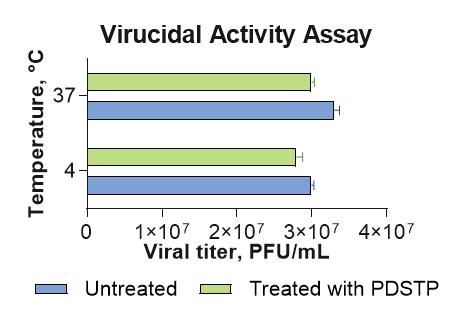


**Figure S2. HPLC traces for PDSTP**

Peak Results

| **#** | **RT, min** | **Peak Height, mAU** | **Peak Area, mAu·sec** | **Asymmetry** |
| --- | --- | --- | --- | --- |
| 1 | 6.955 | 206.39 | 10329 | 0.86 |

**Figure S3**. Map of plasmid pUC57-N containing the SARS-CoV-2 N gene


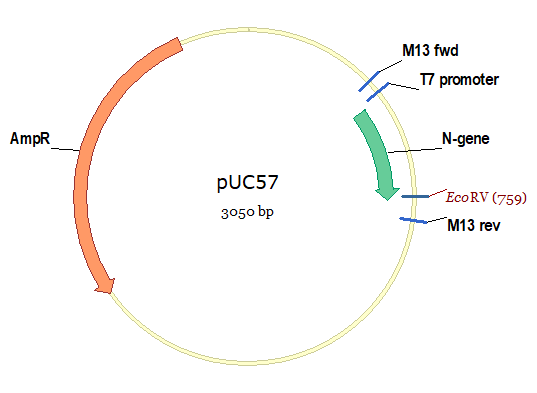


**Figure S4**. RT-qPCR standard curve for calculating RNA copy number using a Bio-Rad CFX manager software (Bio-Rad Laboratories, Inc., Hercules, CA USA)


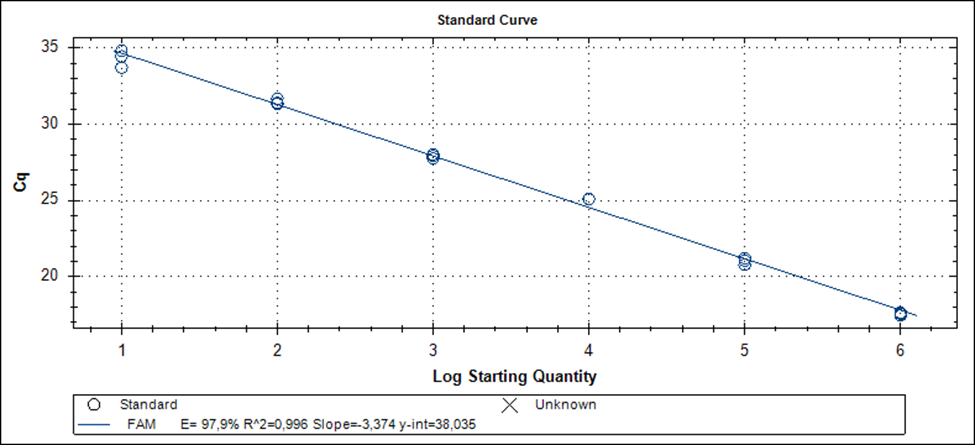


**Tables**

**Table S1. Cytotoxicity and *in vitro* activity of selected dispirotripiperazines against the OC43 strain by the MTT test**

| **Number** | **Chemical structure** | **CC_50_ (Vero-76), µM** | **IC_50_ (HCoV-OC43), µM** |
| --- | --- | --- | --- |
| **PDSTP** |  | **>100.0** | **0.7±0.4** |
| 11826228 |  | >100.0 | 8.5±2.0 |
| 11926056 |  | >100.0 | 6.0±2.0 |
| 11826229 |  | >100.0 | 65.0±5.0 |
| 11826235 |  | >100.0 | 7.0±2.0 |
| 11926061 |  | >100.0 | 50.0±3.0 |
| 12026132 |  | 280.0 | 6.8±2.0 |
| 12026131 |  | >100.0 | 13.0±0.8 |
| 12126016 |  | 39.0 | 7.0±0.9 |
| 12026127 |  | >100.0 | 13.0±3.0 |
| 12026126 |  | >100.0 | 33.0±1.5 |
| 12026128 |  | 100.0 | 7.0±1.6 |
| Remdesivir | | >100.0 | - |
| Hydroxychloroquine | | 40.0±5.0 | 9.8±1.7 |

Table S2. Dynamics of body weight of Syrian hamsters in subgroups during 1-7 days after infection with SARS-CoV-2 virus at a dose of 4×104 TCID^50^ (intranasally)

| Group of animals | units | Days post-infection | | | | | | | |
| --- | --- | --- | --- | --- | --- | --- | --- | --- | --- |
|  |  | day 0 | day 1 | day 2 | day 3 | day 4 | day 5 | day 6 | day 7 |
| Control | gram | 89,6±1,9 | 85,6±0,8 | 85,1±1,4 | 83,1±1,3 | 84,0±1,1 | 82,5±2,2 | 81,3±0,3 | 82,1±0,8 |
|  | % of background | - | 95,2±0,7 | 95,0±1,5 | 92,3±0,5 | 93,8±0,9 | 94,1±1,0 | 94,4±0,7 | 94,1±1,8 |
| PDSTP 10 mg/kg | gram | 100,7±0,3 | 99,0±1,2 | 97,7±1,4 | 95,7±1,6 | 95,5±1,7 | 92,9±2,2 | 93,1±2,5 | 94,3±2,6 |
|  | % of background | - | 98,3±0,6 | 96,5±0,8 | 94,4±1,0 | 94,2±1,2 | 92,5±0,6 | 92,5±0,7 | 93,6±1,0 |
| PDSTP 20 mg/kg | gram | 94,1±0,4 | 94,2±1,9 | 93,3±1,0 | 90,8±0,6 | 91,1±1,0 | 91,8±0,9 | 91,9±0,9 | 91,3±1,8 |
|  | % of background | - | 98,9±0,9 | 97,9±2,1 | 96,5±1,1 | 95,9±0,7 | 96,4±1,0 | 96,8±2,0 | 96,0±2,1 |
| PDSTP 40 mg/kg | gram | 96,7±0,3 | 94,4±1,1 | 95,4±1,4 | 93,2±0,9 | 92,5±0,6 | 91,5±0,8 | 90,8±0,9 | 91,1±0,9 |
|  | % of background | - | 96,9±0,9 | 98,1±0,5 | 95,1±0,6 | 94,7±1,2 | 93,7±1,4 | 93,1±1,1 | 94,0±1,2 |

**Summary of toxicity and safety data for PDSTP**

**General toxicity**

- Acute toxicity, intravenous administration:

Rats (M/F) LD_50_ = 139.5 mg/kg,

Mice (M\F) LD_50_ = 150.0 mg/kg.

- Acute toxicity, intraperitoneal administration:

Mice (M/FLD50 = 192 mg/kg.

- Acute toxicity, topical application (mice, rats):

No manifestations of toxicity and local irritant effects were observed when the maximum regulated amount of PDSTP was applied to the skin, LD50 > 2500 mg/kg (greater than 31.25 mg/cm^2^).

- Acute toxicity, intragastric administration (mice, rats):

After administration of PDSTP at maximum doses (1000 mg/kg - mice, 5000 mg/kg - rats), the development of piloerection and diarrhea, deterioration of hair coat, decreased motor activity, and decreased body weight were noted. No changes in body temperature and internal organs of animals were observed. The death of animals was not recorded.

Chronic toxicity, intranasal administration (rats, rabbits)

At repeated administration of PDSTP for 14 days in a wide range of doses (10-200 mg/kg) toxic effect on organs and systems was not revealed.

Conclusion: PDSTP is low toxic compound.

**Mutagenicity and carcinogenicity**

- Ames test

PDSTP doses tested: 0.1 to 1000 µg/ cup.

Test subjects: Salmonella typhimurium strains TA 97, TA 98 and TA 100.

Metabolic activation: MA+ and MA-.

Result: The number of revertant colonies in the MA- and MA+ variants in the solvent control was within the range of variation of the spontaneous level for these strains. The response of the strains to standard mutagens was within normal levels.

Conclusion: PDSTP has no mutagenic activity in the Salmonella/microsome test (Ames test) using Salmonella typhimurium strains TA 97, TA 98 and TA 100 as a test subject.

- Test for accounting for chromosomal aberrations in mouse bone marrow cells

PDSTP doses tested: 400 mg/kg and 1000 mg/kg

Test subjects: F1 hybrids of SWAxC57BL/6 mice.

Method of administration: topically

Result: Aberrations in experimental and control variants were represented by chromatid and isochromatid fragments. The level of chromosome aberrations in the experimental variants did not significantly exceed the corresponding control values.

Conclusion: PDSTP did not reveal the ability to induce chromosomal aberrations in mammalian somatic cells.

- DNA damage accounting test (DNA Comet)

PDSTP doses tested: 400 mg/kg and 1000 mg/kg

Test subjects: F1 hybrids of SWAxC57BL/6 mice.

Method of administration: topically.

Result: The spontaneous level of DNA damage in cells of tested organs (bone marrow, liver, kidney and spleen) of control group and experimental groups (doses of 400 and 1000 mg/kg) showed no statistical differences between them.

Conclusion: PDSTP does not induce DNA damage in bone marrow, liver, kidney and spleen cells of mice.

**Specific toxicity**

- PDSTP allergenicity study

When tested on experimental animals - guinea pigs (intradermal injection of sensitizing dose of PDSTP 40 µg into the ear) - PDSTP has no sensitizing properties in epicutaneous and conjunctival testing.

No immediate and delayed type hypersensitivity reactions were detected.

In the reaction of specific lysis of leukocytes no reliable increase in cell lysis was revealed.

No delayed-type hypersensitivity was detected in SWA x C57BL/6 mice.

PDSTP does not increase the number of eosinophils in the blood of animals, does not affect the phagocytic activity of blood neutrophils.

Conclusion: PDSTP in tests on experimental animals: albino guinea pigs and CBA x C57BL/6 hybrid mice did not show allergenic properties.

- PDSTP immunotoxicity study

PDSTP at long-term topical applications tends to suppress cellular immunity. Immunosuppression was revealed only at exposure to the maximum dose of 100 TD and had a reversible character: 14 days after withdrawal of administration the index of cellular immunity recovered to the level of the index in the negative control.

Conclusion: PDSTP compound has no effect on humoral immunity and functional activity of macrophages when applied topically for 30 days in doses up to 100 TD.

**Reproductive toxicity**

PDSTP doses tested: 10% solution

Test subjects: white laboratory rats of both sexes

- Study of the effect of PDSTP on the generative function of sexually mature animals

The drug was applied topically on 5% of body surface area (3x5 cm area) for 25 days.

Conclusion: no pathological effect of the drug at the studied dose was detected.

- Embryotoxicity study

Pregnant rats from 6 to 19 days of gestation received daily topical application of PDSTP on 5% of the body surface area (3x5 cm area).

Conclusion: PDSTP in the form of 10% solution has no adverse embryotoxic effect. PDSTP does not cause pathologic developmental delay and anatomical malformations of living fetuses.

- Study of the effect of PDSTP on the postnatal development of F1 generation offspring

During the study of postnatal development and fertility of F1 generation offspring born from female rats that received PDSTP from the 6th day of gestation to delivery, the rates of physical development, the rate of maturation of sensory-motor reflexes and emotional-motor behavior, as well as fertility were evaluated in comparison with rats born from intact females.

Conclusion: PDSTP at the studied doses does not adversely affect the studied parameters of rats. No statistically significant effect of PDSTP on fertility of F1 generation offspring was also found.

**References**

1. Sanna G, Madeddu S, Murgia G, Serreli G, Begala M, Caboni P, Incani A, Franci G, Galdiero M, Giliberti G. Potent and Selective Activity against Human Immunodeficiency Virus 1 (HIV-1) of *Thymelaea hirsuta* Extracts. Viruses. 2020; 12(6):664. https://doi.org/10.3390/v12060664.
